# Integrated Analysis Reveals Altered Lipid and Glucose Metabolism and Identifies NOTCH2 as a Biomarker for Parkinson's Disease Related Depression

**DOI:** 10.3389/fnmol.2018.00257

**Published:** 2018-08-31

**Authors:** Mei-Xue Dong, Xia Feng, Xiao-Min Xu, Ling Hu, Yang Liu, Si-Yu Jia, Bo Li, Wei Chen, You-Dong Wei

**Affiliations:** ^1^Department of Neurology, The First Affiliated Hospital of Chongqing Medical University, Chongqing, China; ^2^Department of Neurology, Renmin Hospital of Wuhan University, Hubei General Hospital, Hubei, China; ^3^Department of Neurology, The People's Hospital of Tongliang District, Chongqing, China; ^4^Department of Neurology, The Fifth People's Hospital of Chongqing, Chongqing, China; ^5^Shanghai Applied Protein Technology Co. Ltd., Shanghai, China

**Keywords:** Parkinson's disease, depression, proteomics, metabolomics, integrated analysis, biomarker

## Abstract

Depression is a common comorbidity in Parkinson's disease (PD) but is underdiagnosed. We aim to investigate the altered metabolic pathways of Parkinson's disease-related depression (PDD) in plasma and to identify potential biomarkers for clinical diagnosis. Consecutive patients with PD were recruited, clinically assessed, and patients with PDD identified. Fasting plasma samples were collected from 99 patients and differentially expressed metabolites and proteins between patients with PDD and PD were identified using non-targeted liquid chromatography-mass spectrometry (LC-MS)-based metabolomics and tandem mass tag (TMT)-based proteomics analysis, followed by an integrated analysis. Based on the above results, enzyme-linked immune sorbent assay (ELISA) tests were then performed to identify potential biomarkers for PDD. In clinics, patients with PDD suffered less hypertension and had lower serum low-density lipoprotein cholesterol and apolipoprotein B levels when compared to the other patients with PD. A total of 85 differentially expressed metabolites were identified in metabolomics analysis. These metabolites were mainly lipids and lipid-like molecules, involved in lipid and glucose metabolic pathways. According to proteomics analysis, 17 differentially expressed proteins were identified, and 12 metabolic pathways were enriched, which were predominantly related to glucose metabolism. Integrated analysis indicated that altered lipid and glucose metabolism in PDD may induce cellular injury through oxidative stress. Additionally, plasma levels of several proteins were confirmed to be significantly altered and correlated with depressive severity. NOTCH2 may be a potential blood biomarker for PDD, with an optimal cut-off point of 0.91 ng/ml, a sensitivity value of 95.65%, and a specificity value of 81.58%. Depressive symptoms are associated with lipid and glucose metabolism in patients with PD and NOTCH2 may be a potential blood biomarker for the clinical diagnosis of PDD.

## Introduction

Parkinson's disease (PD) is the second most prevalent neurodegenerative disease following Alzheimer's disease, afflicting about 3.15% of people over 40 years old (Pringsheim et al., 2014). It seriously affects the patient's quality of life, increases mortality rate, and places a heavy economic burden on the family and society (Kowal et al., 2013; Macleod et al., 2014). Although various studies have been conducted to identify diagnostic biomarkers (Sharma et al., 2013), the clinical diagnosis of patients with PD has been primarily based on cardinal motor features, including resting tremor, bradykinesia, and rigidity (Postuma et al., 2015). Nonmotor symptoms, such as constipation (Adams-Carr et al., 2016), hyposmia (Ponsen et al., 2009), rapid eye movement behavior disorder, cognition (Aarsland et al., 2005), anxiety (Broen et al., 2016), and depression (Reijnders et al., 2008) are also reportedly more prevalent in patients with PD. This is probably due to the alteration of extensive neurotransmitters and brain regions in patients with PD (Kalia and Lang, 2015). Those nonmotor symptoms are often more problematic and distressing than the cardinal motor symptoms.

Depression is one of the most prevalent nonmotor symptoms in PD patients, with about 17% of patients with PD suffering from major depressive disorder (Reijnders et al., 2008). Depression can exacerbate motor symptoms, induce further cognitive disorders, deteriorate the quality of life, and increase the suicide rate in patients with PD (Gustafsson et al., 2015). Worse still, depression in patients with PD is underdiagnosed and under-treated, with only approximately 20% of those diagnosed actually receiving treatments (Goodarzi et al., 2016). Depression can be easily ignored as its characteristic symptoms, including loss of appetite and energy, sleep disturbances, and fatigue, overlap with other symptoms in patients with PD (Marsh, 2013). The underdiagnosis of depression in patients with PD can lead to poor outcomes for patients and caregivers. Currently, the diagnosis of depression in patients with PD is based on a clinical interview (Goodarzi et al., 2016), and an accurate and objective way is highly desirable for clinicians. In addition, treatments for the diagnosis of depression in patients with PD are based on clinical interviews (Goodarzi et al., 2016). Similarly, patients with definitive Parkinson's disease-related depression (PDD) also should be individualized and multidimensional (Marsh, 2013), while the underlying pathogenesis of PDD is complex and remains unclear (Sharma et al., 2013).

Various omics data can extend the understanding of many diseases and help us make more discoveries (Campbell et al., 2017). Novel bioinformatics methods further enable integrated perspectives of pathogeneses and identify clinical biomarkers (Berger et al., 2013; Mostafa et al., 2016). Metabolomics is a comprehensive evaluation of total endogenous metabolites while proteomics expands the understanding of a biological system at the protein level (Altelaar et al., 2013; Wishart, 2016). Herein we employed non-targeted liquid chromatography-mass spectrometry (LC-MS)-based metabolomics analysis and tandem mass tag (TMT)-based proteomics analysis to investigate the plasma changes of PDD. An integrated analysis was further generated to elucidate the probable pathogenesis. Based on the above results, enzyme-linked immune sorbent assay (ELISA) tests were then performed to identify potential biomarkers for PDD. In clinical settings, plasma is a relatively accessible, stable, and informative biofluid, making it ideal for exploring the underlying pathogenesis, and facilitating the diagnosis of PDD (Hu et al., 2016; Dong et al., 2017c).

## Materials and methods

### Patients

One hundred and ten consecutively recruited patients with PD were included in the Department of Neurology, the First Affiliated Hospital of Chongqing Medical University from April 2016 to February 2017 in accordance with the following inclusion criteria: (i) Parkinson's disease diagnosed according to the recommendation from the European Federation of Neurological Societies (EFNS) and the International Parkinson Movement Disorder Society's European Section (MDS-ES) (Berardelli et al., 2013); (ii) Patients not treated with any drugs other than dopamine analogs or dopamine receptor agonists. Eleven patients were then excluded according to the following criteria: (i) secondary Parkinson disorders or Parkinson-plus syndrome; (ii) coexistence of severe systemic diseases (e.g., tumor, chronic heart failure, chronic obstructive pulmonary disease, hepatitis, and nephritis) or infectious diseases at the time of enrolment; (iii) history of stroke, brain surgery or injury, Alzheimer's disease, motor neuron disease, or other diseases in the central nervous system (Dong et al., 2017c).

This study was approved by the ethics committee of the First Affiliated Hospital of Chongqing Medical University and performed in accordance with the Declaration of Helsinki. All subjects provided written informed consent prior to inclusion in this study. Clinical characteristics, metabolomics analysis, and proteomics analysis were blindly assessed or performed.

### Clinical characteristics

The clinical characteristics of all included patients were recorded. Fasting plasma samples were obtained by centrifugation at 2,000 × *g* for 10 min at 4°C after collection with EDTA-K2 tube at 6:00 a.m. by puncture of the median cubital vein. Samples were then stored at −80°C until assay. All patients were interviewed by experienced physicians and patients with PDD were diagnosed according to Diagnostic and Statistical Manual of Mental Disorders (DSM-IV) (Starkstein et al., 2008), with Hamilton depression scores >17 on the 17-item Hamilton Rating Scale for Depression (HAMD) (Dissanayaka et al., 2007). All scales were independently assessed by two physicians.

### Metabolomics analysis

Liquid chromatography-mass spectrometry (LC-MS)-based metabolomics of plasma samples (23 patients with PDD and 40 randomly selected patients with PD) were performed on a Waters UPLC I-class system equipped with a binary solvent delivery manager (Waters Corporation, Milford, USA) (Zhang et al., 2017a).

Plasma samples stored at −80°C were gradually thawed on ice, 2-chloro-1-phenylalanine dissolved in methanol (0.3 mg/mL) was served as internal standard. In a 1.5 mL Eppendorf tube, 50 μL of sample and 10 μL of internal standard were added and then vortexed for 10 s. Subsequently, 150 μL of ice-cold mixture of methanol and acetonitrile (2/1, vol/vol) were added. The mixtures were vortexed for 1 min, ultrasonicated at ambient temperature (25°C) for 5 min, placed at −20°C for 10 min, and centrifuged at 15,000 rpm at 4°C for 10 min. 100 μL of the supernatants from each tube were collected, filtered through 0.22 μm microfilters, and transferred to LC vials. The vials were stored at −80°C until LC-MS analysis. Quality control sample was obtained by mixing all the samples equally as a pooled sample, and then processed using the above method with the analytic reagents. The quality control samples were injected at regular intervals (every 10 samples) throughout the analytical run to provide a set of data from which repeatability can be assessed.

Acquity BEH C18 column (100 × 2.1 mm i.d., 1.7 μm; Waters, Milford, USA) was engaged and maintained at 45°C. The following gradients were used for separation: 5–20% B over 0–2 min, 20–60% B over 2–8 min, 60–100% B over 8–12 min, 100% B for 2 min, 100–5% Bover 14–14.5 min, and 14.5–15.5 min holding at 5% B at a flow rate of 0.40 mL/min, where B is acetonitrile (0.1% (v/v) formic acid) and A is aqueous formic acid [0.1% (v/v) formic acid]. Injection volume was 3.00 μL and column temperature was set at 45°C.

The mass spectrometric data was collected using a Waters VION IMS Q-TOF Mass Spectrometer equipped with an electrospray ionization (ESI) source operating in either positive or negative ion mode. The source temperature and desolvation temperature were set at 120°C and 500°C, respectively, with a desolvation gas flow of 900 L/h. Centroid data were collected from 50 to 1,000 m/z with a scan time of 0.1 s and interscan delay of 0.02 s over a 13 min analysis time. The UPLC-Q-TOF/MS raw data were analyzed by progenesis QI software (Waters Corporation, Milford, USA) using the following parameters. Retention time (RT) ranged from 0.5 to 14.0 min, mass ranged from 50 to 1,000 Da, and mass tolerance was 0.01 Da. Isotopic peaks were excluded for analysis, noise elimination level was set at 10.00, minimum intensity was set to 15% of base peak intensity, and RT tolerance was set at 0.01 min. Three-dimension data sets including m/z, peak RT, and peak intensities were exported into an Excel file, and RT–m/z pairs were used to identify each ion based on Human Metabolome Database (HMDB, http://www.hmdb.ca), Metlin (https://metlin.scripps.edu), and LipidMaps (http://www.lipidmaps.org). The resulting matrix was further reduced by removing any peaks with missing values (ion intensity = 0) in more than 60% of samples. The internal standard was used for data quality control.

The positive and negative data were combined into a data set and imported into SIMCA-P+ 13.0 software package (Umetrics, Umeå, Sweden) for multivariate statistical analysis. An orthogonal partial least squares-discriminant analysis (OPLS-DA) model was used to exhibit statistical differences and identify differentially expressed metabolites in patients with PDD relative to the other patients with PD, and this model was validated by a permutation test with 200 iterations. The differentially expressed metabolites were recognized with variable influence on projection values of greater than 1.0, fold change values of greater than ±1.5, and *p*-values (from Mann-Whitney *U*-tests) of less than 0.05 (Zheng et al., 2016; Dong et al., 2017b). The classifications of differentially expressed metabolites were based on the HMDB database. Metabolic pathway overrepresentation enrichment analysis was also performed using Integrated Molecular Pathway Level Analysis (http://impala.molgen.mpg.de) (Kamburov et al., 2011). Q values were calculated by Benjamini-Hochberg correction for multiple testing to adjust the derived *p* values and significances were considered at *q*-values < 0.05 (Hochberg and Benjamini, 1990).

### Proteomics analysis

The details of this process have been described previously (Dayon et al., 2014; Sogawa et al., 2016). Briefly, six pooled samples were obtained by the accumulation of 50 μl of each plasma sample (15 randomized patients with PDD and 30 randomized patients with PD were equally grouped into three pooled samples, respectively). Most of the abundant proteins were depleted from those pooled samples using Agilent Human 14 Multiple Affinity Removal System Column (Agilent Technologies, California, USA) following the manufacturer's protocol. The 10 KDa ultrafiltration tube (Sartorius, Göttingen, Germany) was used for desalination and concentration of the low-abundance components. One volume of SDT buffer (4% SDS, 100 mM Tris-HCl, 1mM DTT, pH 7.6) was added. The mixture was boiled for 15 min and centrifuged at 14,000 g for 20 min. The supernatant was quantified with the BCA Protein Assay Kit (Bio-Rad, California, USA). Finally, the sample was stored at −80°C until assay.

For each sample, 20 μg of proteins were mixed with 5X loading buffer and boiled for 5 min. The proteins were then separated on 12.5% SDS-PAGE gel (constant current 14 mA, 90 min) and protein bands were visualized by Coomassie Blue R-250 staining for quality control.

After that, 200 μg of proteins for each sample were incorporated into 30 μl SDT buffer (4% SDS, 100 mM DTT, 150 mM Tris-HCl pH 8.0). The detergent, dithiothreitol (DTT), and other low-molecular-weight components were removed using UA buffer (8 M Urea, 150 mM Tris-HCl pH 8.0) by repeated ultrafiltration. Then 100 μl iodoacetamide (100 mM IAA in UA buffer) was added to block reduced cysteine residues and the samples were incubated for 30 min in darkness. The filters were washed with 100 μl UA buffer three times and then 100 μl 100 mM TEAB buffer twice. Finally, the protein suspensions were digested with 4 μg trypsin (Promega, Wisconsin, USA) in 40 μl TEAB buffer overnight at 37°C, and the resulting peptides were collected as a filtrate. The peptide content was estimated by ultraviolet light (280 nm) using an extinction coefficient of 1.1 of 0.1% (g/l) solution that was calculated based on the frequency of tryptophan and tyrosine in vertebrate proteins.

Using TMT reagents according to the manufacturer's instructions, 100 μg peptide mixture of each sample was labeled (Thermo Scientific, Massachusetts, USA). A Pierce high pH reverse-phase fractionation kit (Thermo Scientific) was used to fractionate TMT-labeled digest samples into 15 fractions by an increasing acetonitrile step-gradient elution carried according to the instructions.

Each fraction was loaded onto a reverse phase trap column connected to the C18-reversed phase analytical column in buffer A (0.1% Formic acid) and separated with a linear gradient of buffer B (84% acetonitrile and 0.1% Formic acid) at a flow rate of 300 nl/min controlled by IntelliFlow technology (Thermo Scientific) for nano LC-MS/MS analysis.

Liquid chromatography-mass spectrometry/MS (LC-MS/MS) analysis was performed on a Q Exactive mass spectrometer (Thermo Scientific) that was coupled to Easy nLC for 60 min. The mass spectrometer was operated in positive ion mode. Mass spectrometric data was acquired using a data-dependent top 10 method dynamically by choosing the most abundant precursor ions from the survey scan (300–1,800 m/z) for HCD fragmentation. Automatic gain control (AGC) target was set to 3E6 and maximum inject time to 10 min. Dynamic exclusion duration was 40 s. Survey scans were acquired at a resolution of 70,000 at 200 m/z and resolution for HCD spectra was set to 35,000 at 200 m/z, and isolation width was 2 m/z. Normalized collision energy was 30 eV and the underfill ratio, which specifies the minimum percentage of the target value likely to be reached at maximum fill time, was defined as 0.1%. The instrument was run with the peptide recognition mode enabled.

Tandem mass spectrometry (MS/MS) spectra were searched using MASCOT engine (Matrix Science, London, UK; version 2.2) embedded into Proteome Discoverer 1.4. The differentially expressed proteins were identified by fold change values of greater than ±1.2 and *p* < 0.05 (from Mann-Whitney *U*-tests). Gene ontology (GO) enrichment on three ontologies (biological process, molecular function, and cellular component) and Kyoto Encyclopedia of Genes and Genomes (KEGG) pathway enrichment analyses were applied based on the Fisher's exact test, considering the whole quantified protein annotations as background dataset. Benjamini-Hochberg correction for multiple testing was further applied to adjust derived *p* values and significances were considered at *q*-values < 0.05 (Hochberg and Benjamini, 1990).

### Integrated analysis

To explore the metabolic pathways associated with the differentially expressed metabolites and proteins, we used the commercially obtained Ingenuity Pathway Analysis software (IPA, QIAGEN, Düsseldorf, Germany) to annotate enriched molecular or cellular functions and to generate metabolite-protein integrated networks (Dong et al., 2017b; Shen et al., 2017). We uploaded the lists and fold change values of differentially expressed metabolites and proteins onto the IPA software. Significant molecular or cellular functions were automatically annotated (*p* < 0.05) and enriched into several categories. The software also computed a *p* score for each of the possible networks in accordance with the fit homology to all the input molecules. This score is derived from a *p* value and indicates the probability of the input molecules in a given network to coexist as a result of random chance [*p* score = –log_10_ (*p* value)].

### ELISA tests

According to the above network from integrated analysis, plasma levels of six differentially expressed proteins [receptor-type tyrosine-protein phosphatase zeta (PTPRZ1), major histocompatibility complex class I antigen (HLA-A), neurogenic locus notch homolog protein 2 (NOTCH2), lipoprotein (LPA), L-lactate dehydrogenase A chain (LDHA), and glyceraldehyde-3-phosphate dehydrogenase (GAPDH)] identified by proteomics analysis were further confirmed in 23 patients with PDD and 76 patients with PD, using ELISA kits obtained from Cloud Clone Corp (Texas, USA) according to the manufacturer's instructions (Hu et al., 2016).

### Statistical analysis

Statistical analyses were completed using a commercially available software package (IBM SPSS version 22.0, New York, USA), with statistical diagrams produced using GraphPad Prism 5 (GraphPad Software, California, USA) (Dong et al., 2017a,c). Continuous data were expressed as mean ± standard errors of the mean and compared using Mann-Whitney *U*-tests. Categorical data were exhibited as absolute numbers and percentages (%) and analyzed using Pearson χ^2^-tests or Fisher exact tests. Pearson correlation analysis between plasma protein levels and HAMD scores were performed and plotted using Cytoscape software 3.4.0 (Zhao et al., 2017). Receiver operating characteristic (ROC) curves were plotted, the optimal cut-off points were determined, and diagnostic efficacies were compared using MedCalc statistical software 15.2.2 (Ostend, Belgium). *P*-values < 0.05 were considered significant (Dong et al., 2016).

## Results

### Clinical characteristics

A total of 99 patients with PD were finally included after the exclusion of 11 patients. Depressive symptoms were present in 23 patients (23.2%), which was approximately equal to the reported 17% in the literature (Reijnders et al., 2008). The clinical characteristics of the patients with PD are shown in Table 1. The 17-item HAMD scores of patients with PDD were significantly higher than those in patients with PD. Patients with PDD also suffered less hypertension and had lower serum low-density lipoprotein cholesterol (LDL-C) and apolipoprotein B (Apo-B) levels than the patients with PD. There were no significant differences in any other clinical characteristics, including the levels of blood glucose and hemoglobin A1C (HbA1C) and incidence of diabetes mellitus.

**Table 1:** Clinical characteristics of all patients with PD with and without depression included in this study.

**Variable (SEM/%)**	**PD (76)**	**PDD (23)**	***p* value**	**Variable (SEM/%)**	**PD (76)**	**PDD (23)**	***p* value**
Age (year)	68.92 ± 1.04	66.48 ± 2.16	0.273	RBG (mmol/L)	7.01 ± 0.24	7.28 ± 0.62	0.623
Gender, Male (%)	44 (57.9%)	10 (43.5%)	0.224	HbA1C (%)	5.97 ± 0.09%	7.09 ± 0.85%	0.230
Smoking history (%)	15 (19.7%)	2 (8.7%)	0.360	TC (mmol/L)	3.95 ± 0.18	3.70 ± 0.29	0.511
Alcohol consumption (%)	7 (9.2%)	1 (4.3%)	0.754	TG (mmol/L)	1.24 ± 0.09	1.12 ± 0.16	0.498
Hypertension (%)	31 (40.8%)	4 (17.4%)	0.040	HDL-C (mmol/L)	1.38 ± 0.05	1.49 ± 0.07	0.261
Diabetes mellitus (%)	9 (11.8%)	4 (17.4%)	0.735	LDL-C (mmol/L)	2.78 ± 0.09	2.37 ± 0.15	0.032
Hypercholesterolemia (%)	16 (21.1%)	2 (8.7%)	0.299	Apo-A1 (g/L)	1.33 ± 0.03	1.38 ± 0.04	0.422
CHD (%)	12 (15.8%)	4 (17.4%)	1.000	Apo-B (g/L)	0.88 ± 0.03	0.74 ± 0.04	0.010
Disease duration (year)	5.62 ± 0.63	7.35 ± 0.93	0.163	HAMD score	8.96 ± 0.61	21.48 ± 0.57	0.000
BMI (kg/m^2^)	23.11 ± 0.46	22.25 ± 0.67	0.340				

*PD, Parkinson's disease; SEM, standard error of the mean; PDD, Parkinson's disease related depression; RBG, random blood glucose; HbA1C, hemoglobin A1C; TC, total cholesterol; TG, triglyceride; HDL-C, high-density lipoprotein cholesterol; LDL-C, low-density lipoprotein cholesterol; Apo-A1, apolipoprotein A1; CHD, coronary heart disease; Apo-B, apolipoprotein B; HAMD, Hamilton Depression Scale; BMI, body mass index*.

### Metabolomics analysis

**Figure 1:**
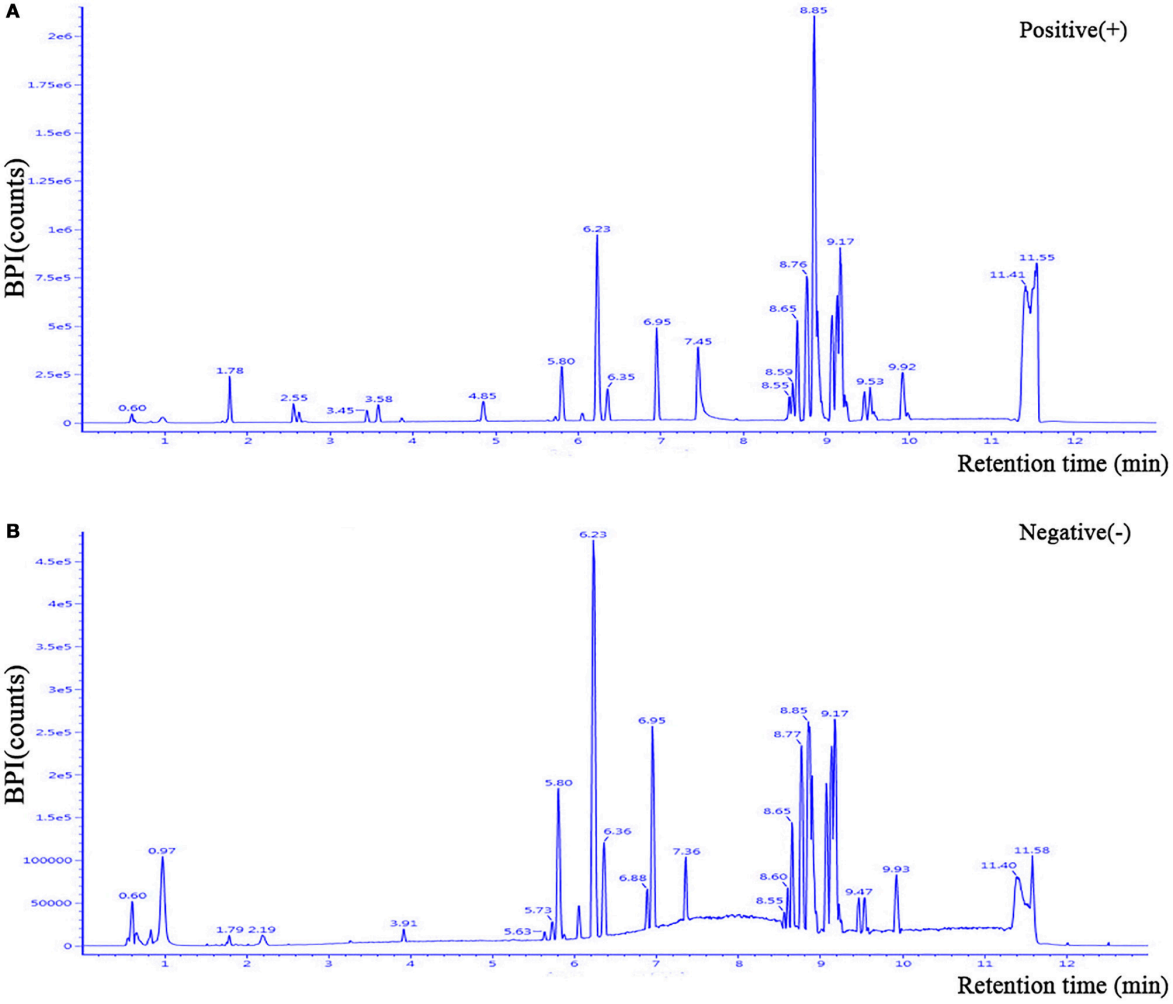
Representative LC-MS positive **(A)** and negative **(B)** ions of current chromatograms of plasma in a patient with PDD. LC-MS, liquid chromatography-mass spectrometry.

**Figure 2:**
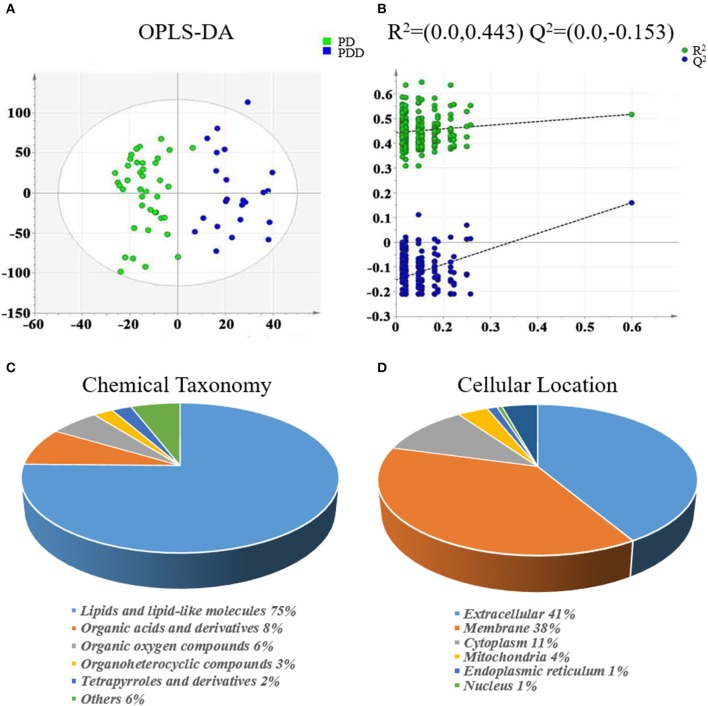
Multivariate statistical analysis of metabolomics and classification of differentially expressed metabolites between patients with PDD and PD. **(A)** OPLS-DA score plot derived from liquid chromatography-mass spectrometry-based metabolomics analysis of patients with PDD (blue circles) and patients with PD (green circles). **(B)** Statistical validation of the OPLS-DA model by permutation testing. Representations of metabolites in terms of **(C)** chemical taxonomy and **(D)** cellular locations, based on the annotations of Human Metabolome Database. OPLS-DA, orthogonal partial least squares-discriminant analysis; PDD, Parkinson's disease-related depression; PD, Parkinson's disease.

The clinical characteristics of the patients included in the LC-MS-based metabolomics analysis are shown in Supplementary Table 1 and the results were comparable with the total patient group. Representative LC-MS positive and negative ions of current chromatograms are shown in Figure 1. After excluding internal standards, a total of 10403 individual peaks, including 6040 positive and 4363 negative peaks, were detected in approximately 98.8% of samples in each group. Based on these peaks, orthogonal partial least squares-discriminant analysis (OPLS-DA) was performed and the result showed a clear separation between the two groups (R^2^X = 0.389, R^2^Y = 0.832, and *Q*^2^ = 0.206) (Figure 2A). Moreover, a permutation test with 200 iterations confirmed that the constructed OPLS-DA model was valid and not over-fitted, as the original right *R*^2^ and *Q*^2^ values were significantly higher than the corresponding permutated left values [*R*^2^ = (0.0, 0.443), *Q*^2^ = (0.0, −0.153); Figure 2B].

Eighty-five differentially expressed metabolites were identified between the two groups (Table 2). Of these 75% were lipids and lipid-like molecules, 8% were organic acids and derivatives, 6% were organic oxygen compounds, 3% were organoheterocyclic compounds, and 2% were tetrapyrroles and derivatives (Figure 2C). Carbohydrates and carbohydrate conjugates were a subclass of organic oxygen compounds and contained four differentially expressed metabolites ([6]-gingerdiol 4′-O-beta-D-glucopyranoside, ribitol, galactan, and 1-(alpha-Methyl-4-(2-methylpropyl)benzeneacetate)-beta-D-Glucopyranuronic acid). Those differentially expressed metabolites were primarily located in the extracellular space (41%), membrane (38%), cytoplasm (11%), mitochondria (4%), endoplasmic reticulum (1%), and nucleus (1%) (Figure 2D).

**Table 2:** Key differentially expressed metabolites identified by liquid chromatography-mass spectrometry-based metabolomics analysis between patients with PD with and without depression.

**Compound ID^a^**	**Compound name**	**m/z value**	**RT (min)**	**Ion mode**	**FCvalue^b^, ^c^**	**VIPvalue^c^**	***p*value^c^**
HMDB36133	3-[[5-methyl-2-(1-methylethyl)cyclohexyl]oxy]-1,2-propanediol	275.1573	6.6012	Positive	0.6558	1.7468	0.0000
HMDB31892	Capsianoside II	362.5188	6.2251	Positive	0.6412	1.1183	0.0011
HMDB02327	1,11-undecanedicarboxylic acid	511.3255	6.2251	Positive	0.6114	1.3346	0.0030
HMDB56318	DG(20:2n6/0:0/20:5n3)	349.2424	7.8697	Positive	2.4783	1.1013	0.0038
HMDB02472	(3a,5b,7a,12a)-24-[(carboxymethyl)amino]-1,12-dihydroxy-24-oxocholan-3-yl-b-D-glucopyranosiduronic a	622.3352	6.4951	Negative	0.5919	1.3399	0.0038
HMDB09112	PE(18:2(9Z,12Z)/24:0)	291.6477	6.7939	Positive	0.5684	1.2768	0.0039
HMDB11768	Cer(d18:0/24:0)	564.3164	6.3557	Positive	0.6219	1.1326	0.0043
HMDB12052	Ganglioside GT3	849.9375	6.2251	Positive	0.5873	1.0936	0.0046
HMDB02231	Eicosenoic acid	309.2803	7.8649	Negative	1.7393	1.1741	0.0053
5283546	Ubiquinone 8	707.5316	9.3385	Negative	0.5691	1.5228	0.0055
HMDB61687	Estradiol acetate glucuronide	491.2330	7.8697	Positive	2.1162	1.0804	0.0062
HMDB07004	CPA(18:0)	457.2138	7.8649	Negative	1.7405	1.3792	0.0066
HMDB05050	15H-11,12-EETA	717.4415	9.3385	Negative	0.6480	1.3399	0.0067
HMDB35884	Melledonol	435.2033	7.8697	Positive	1.8629	1.1410	0.0070
HMDB10393	LysoPC(20:3(5Z,8Z,11Z))	580.3185	6.0620	Negative	0.6378	1.1149	0.0072
HMDB00054	Bilirubin	602.2940	6.9452	Positive	0.6526	1.1481	0.0072
HMDB61112	3-carboxy-4-methyl-5-propyl-2-furanpropionic acid	503.1898	7.8697	Positive	1.6347	1.1415	0.0073
HMDB06528	Docosapentaenoic acid	329.2489	7.0345	Negative	1.5535	1.2833	0.0079
HMDB13132	Hydroxyvalerylcarnitine	571.3428	6.6101	Negative	0.6668	1.5142	0.0085
HMDB36122	[6]-gingerdiol 4′-O-beta-D-glucopyranoside	439.2376	7.8649	Negative	2.3428	1.3651	0.0092
HMDB10357	Tetrahydroaldosterone-3-glucuronide	539.2509	2.8699	Negative	0.5838	1.4905	0.0098
HMDB03876	15(S)-hydroxyeicosatrienoic acid	367.2137	7.8628	Positive	1.6883	1.2439	0.0099
HMDB03178	Heme	661.1760	7.8718	Negative	2.2621	1.4164	0.0101
HMDB04924	Ganglioside GD2 (d18:0/18:1(9Z))	856.9514	6.2251	Positive	0.5699	1.3039	0.0101
HMDB33372	Stearoyllactic acid	377.2690	7.8649	Negative	1.6552	1.2258	0.0101
HMDB04666	2-arachidonylglycerol	417.2318	7.8628	Positive	1.6767	1.0197	0.0111
HMDB00207	Cis-cetoleic acid	337.3120	8.3029	Negative	2.0741	1.7107	0.0126
HMDB40733	Phytyl acetate	383.2901	8.3010	Positive	2.6419	1.6090	0.0144
HMDB01181	4α-carboxy-4β-ethyl-5α-cholesta-8,24-dien-3β-ol	519.2643	8.3010	Positive	2.6728	1.6980	0.0145
HMDB02385	Celastrol	485.2448	8.3029	Negative	2.3661	1.5956	0.0155
HMDB00235	Thiamine	531.2198	8.3010	Positive	2.3720	1.6565	0.0157
HMDB06765	2-methoxy-estradiol-17b 3-glucuronide	459.2057	7.8718	Negative	1.5666	1.1844	0.0157
HMDB33337	Fusarin C	468.1464	6.4264	Negative	1.7382	1.4644	0.0168
HMDB00809	N-glycoloylganglioside GM1	812.9456	6.2251	Positive	0.6698	1.2230	0.0177
LMPR04000010	2-methylbacteriohopane-32,33,34,35-tetrol	581.4554	6.8783	Negative	1.5976	1.2967	0.0178
HMDB00651	Decanoylcarnitine	653.4745	8.6022	Positive	0.6570	1.1854	0.0180
HMDB06246	Tetracosatetraenoic acid (24:4n-6)	405.2998	8.3029	Negative	2.3812	1.6977	0.0186
HMDB04309	Triterpenoid	535.2935	6.0458	Positive	0.6488	1.3223	0.0189
HMDB11494	LysoPE(0:0/22:5(4Z,7Z,10Z,13Z,16Z))	526.2952	5.2339	Negative	0.4995	1.1323	0.0200
HMDB00508	Ribitol	455.1960	7.4471	Negative	1.8272	1.0417	0.0202
HMDB00027	Tetrahydrobiopterin	500.2783	5.3003	Positive	0.5117	1.2442	0.0210
HMDB02925	8,11,14-eicosatrienoic acid	339.2892	9.3361	Positive	0.6451	1.1500	0.0220
HMDB29482	Didymin	593.1895	7.8649	Negative	1.9325	1.2976	0.0224
HMDB09055	PE(18:1(9Z)/16:0)	794.4496	6.2251	Positive	0.6046	1.2594	0.0228
HMDB53219	TG(18:3(9Z,12Z,15Z)/20:5(5Z,8Z,11Z,14Z,17Z)/20:5(5Z,8Z,11Z,14Z,17Z))[iso3]	901.6793	7.3508	Negative	1.6115	1.3523	0.0228
HMDB61701	2-oleoylglycerophosphocholine	521.3495	5.5826	Negative	0.6010	1.3357	0.0232
LMST05010016	6alpha-glucuronosylhyodeoxycholate	284.6664	6.0595	Positive	0.6197	1.2900	0.0233
HMDB00476	3-oxo-4,6-choladienoic acid	779.4714	6.2251	Positive	0.5820	1.3608	0.0234
HMDB08189	PC(18:3(6Z,9Z,12Z)/22:6(4Z,7Z,10Z,13Z,16Z,19Z))	850.5363	8.2253	Positive	0.4798	1.1905	0.0237
HMDB00387	3-hydroxydodecanoic acid	293.0903	2.1975	Positive	0.5924	1.4002	0.0243
HMDB60579	Ramiprilat	425.1488	6.4264	Negative	1.5224	1.3263	0.0246
HMDB00913	Vanillactic acid	469.1365	6.6995	Negative	2.0884	1.2691	0.0249
HMDB10326	Thyroxine glucuronide	951.6940	7.3508	Negative	1.7267	1.3201	0.0250
HMDB00949	Tetrahydrocortisol	755.4739	7.4452	Positive	1.7720	1.2388	0.0263
HMDB03577	VPGPR Enterostatin	557.3389	6.9452	Positive	0.5808	1.1159	0.0264
HMDB04913	Ganglioside GD3 (D18:1/16:0)	804.9525	6.2251	Positive	0.6007	1.3974	0.0266
HMDB60508	Secalciferol	877.6602	6.8783	Negative	2.1024	1.4062	0.0273
HMDB03598	Retinyl ester	649.4431	6.8783	Negative	1.5325	1.2533	0.0278
HMDB34336	Dolichosterone	969.6667	7.3508	Negative	1.6119	1.3317	0.0283
HMDB11874	Ganglioside GD3 (d18:1/23:0)	793.9482	6.2251	Positive	0.6681	1.4021	0.0286
HMDB35864	13-hydroxy-5′-O-methylmelledonal	232.1028	0.6080	Positive	0.4595	1.1234	0.0294
HMDB55312	TG(18:4(6Z,9Z,12Z,15Z)/18:4(6Z,9Z,12Z,15Z)/20:3(8Z,11Z,14Z))[iso3]	941.6378	7.3489	Positive	1.7494	1.3972	0.0294
HMDB10397	LysoPC(20:5(5Z,8Z,11Z,14Z,17Z))	542.3235	5.2315	Positive	0.5336	1.2340	0.0298
HMDB13456	PC(O-22:2(13Z,16Z)/22:3(10Z,13Z,16Z))	878.7119	7.3489	Positive	2.2710	1.0600	0.0316
HMDB09210	PA(18:4(6Z,9Z,12Z,15Z)/22:6(4Z,7Z,10Z,13Z,16Z,19Z))	741.4523	7.8628	Positive	1.9090	1.1151	0.0320
HMDB00079	Dihydrothymine	383.1757	6.6995	Negative	1.6348	1.1711	0.0329
HMDB13111	Ubiquinol-10	941.6087	6.8764	Positive	1.7289	1.3385	0.0335
HMDB04162	Galactan	533.2137	8.3010	Positive	1.7240	1.5959	0.0340
HMDB07046	DG(14:1(9Z)/18:3(6Z,9Z,12Z)/0:0)	605.1058	6.6995	Negative	2.2738	1.3274	0.0341
HMDB02596	Deoxycholic acid 3-glucuronide	607.2992	6.9452	Positive	0.6623	1.0737	0.0356
HMDB13622	Nonadeca-10(Z)-enoic acid	295.2644	7.6170	Negative	1.7845	1.3472	0.0357
HMDB61691	1-heptadecanoylglycerophosphoethanolamine	935.5914	6.8764	Positive	1.9111	1.3195	0.0373
HMDB01185	S-adenosylmethionine	354.1748	6.6976	Positive	1.8280	1.2410	0.0378
HMDB10343	1-(alpha-Methyl-4-(2-methylpropyl)benzeneacetate)-beta-D-Glucopyranuronic acid	403.1453	6.6995	Negative	1.7895	1.1716	0.0409
HMDB07892	PC(14:0/22:6(4Z,7Z,10Z,13Z,16Z,19Z))[U]	822.5290	8.2685	Negative	0.5697	1.2126	0.0425
HMDB01999	Eicosapentaenoic acid	301.2182	8.5553	Negative	0.4157	1.0496	0.0427
HMDB04863	Ganglioside GM1 (d18:1/24:0)	825.0025	6.9452	Positive	0.5165	1.2551	0.0442
HMDB11489	LysoPE(0:0/20:5(5Z,8Z,11Z,14Z,17Z))	498.2638	5.3026	Negative	0.5686	1.2098	0.0463
HMDB11891	Ganglioside GM1 (d18:1/18:1(11Z))	513.2805	5.1514	Negative	2.6454	1.0136	0.0468
HMDB01138	N-acetylglutamic acid	379.1386	6.6976	Positive	1.6936	1.2277	0.0473
HMDB32033	2,4,12-octadecatrienoic acid isobutylamide	378.2729	9.0679	Positive	0.6475	1.0131	0.0474
HMDB00962	Lipoamide	586.3166	5.0825	Negative	0.4844	1.1850	0.0479
HMDB00319	18-hydroxycorticosterone	395.2425	8.3010	Positive	1.6211	1.6175	0.0484
HMDB07951	PC(15:0/20:5(5Z,8Z,11Z,14Z,17Z))	810.5286	8.4060	Negative	0.4467	1.0508	0.0489
HMDB32002	Dehydrotomatine	352.1781	6.6976	Positive	1.5554	1.1068	0.0492

a *Compound ID was mainly exhibited based on the Human Metabolome Database (www.hmdb.ca), and the others based on National Center for Biotechnology Information (www.ncbi.nlm.nih.gov/pccompound/) or LIPID MAPS (www.lipidmaps.org)*.

b *FC value was calculated as the ratio of the average mass response (area) between the two groups (FC value = PDD/PD). Thus, FC values >1 indicate significantly higher levels in the PDD group relative to the PD group while FC values <1 indicate significantly lower levels in the PDD group*.

c *Only metabolites with FC values greater than ± 1.5, VIP values greater than 1.0 and p values less than 0.05 were deemed statistically significant*.

*PD, Parkinson's disease; RT, retention time; FC, fold change; VIP, variable influence on projection; PDD, Parkinson's disease related depression*.

**Figure 3:**
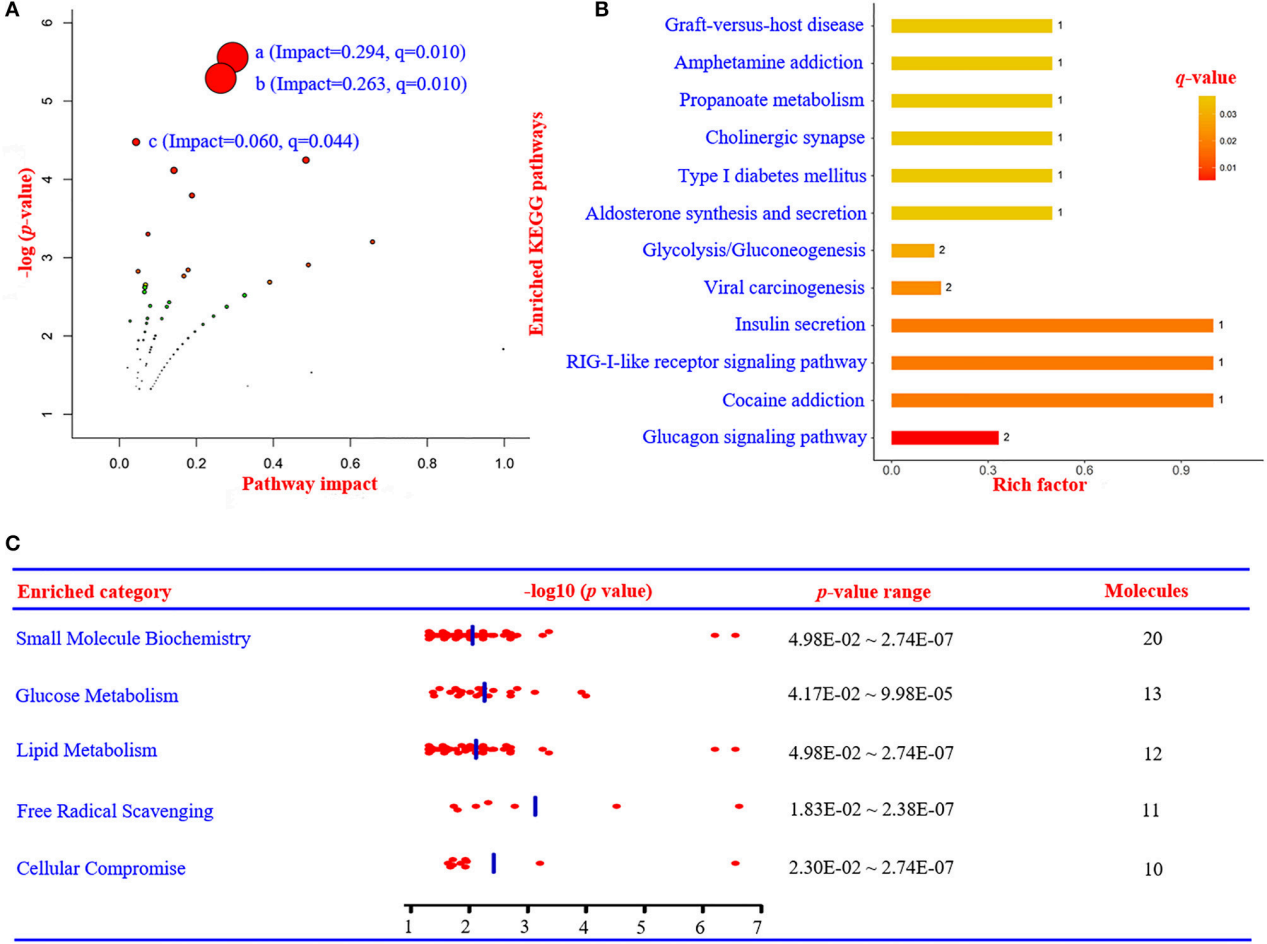
Summary of metabolic pathways based on metabolomics, proteomics, and integrated analysis. **(A)** Metabolic pathway analysis based on metabolomics data using IMPaLA. (a) Alpha-linolenic acid and linoleic acid metabolism (impact = 0.294, *q* = 0.010). (b) Incretin synthesis, secretion, and inactivation (impact = 0.263, *q* = 0.010). (c) Signal transduction (impact = 0.060, *q* = 0.044). **(B)** Enriched KEGG pathways based on proteomics data. **(C)** The most significant enriched function annotations analyzed by Ingenuity Pathways Analysis, based on the differentially expressed metabolites and proteins between patients with PDD and PD. KEGG, Kyoto Encyclopedia of Genes and Genomes; PDD, Parkinson's disease-related depression; PD, Parkinson's disease.

The related metabolic pathways are shown in Figure 3A and Supplementary Table 2, with three most significant pathways: (1) alpha-linolenic acid and linoleic acid metabolism (pathway impact = 0.294, *q*-value = 0.0102); (2) incretin synthesis, secretion, and inactivation (pathway impact = 0.263, *q*-value = 0.0102); and (3) signal transduction (pathway impact = 0.060, *q*-value = 0.0443).

### Proteomics analysis

**Figure 4:**
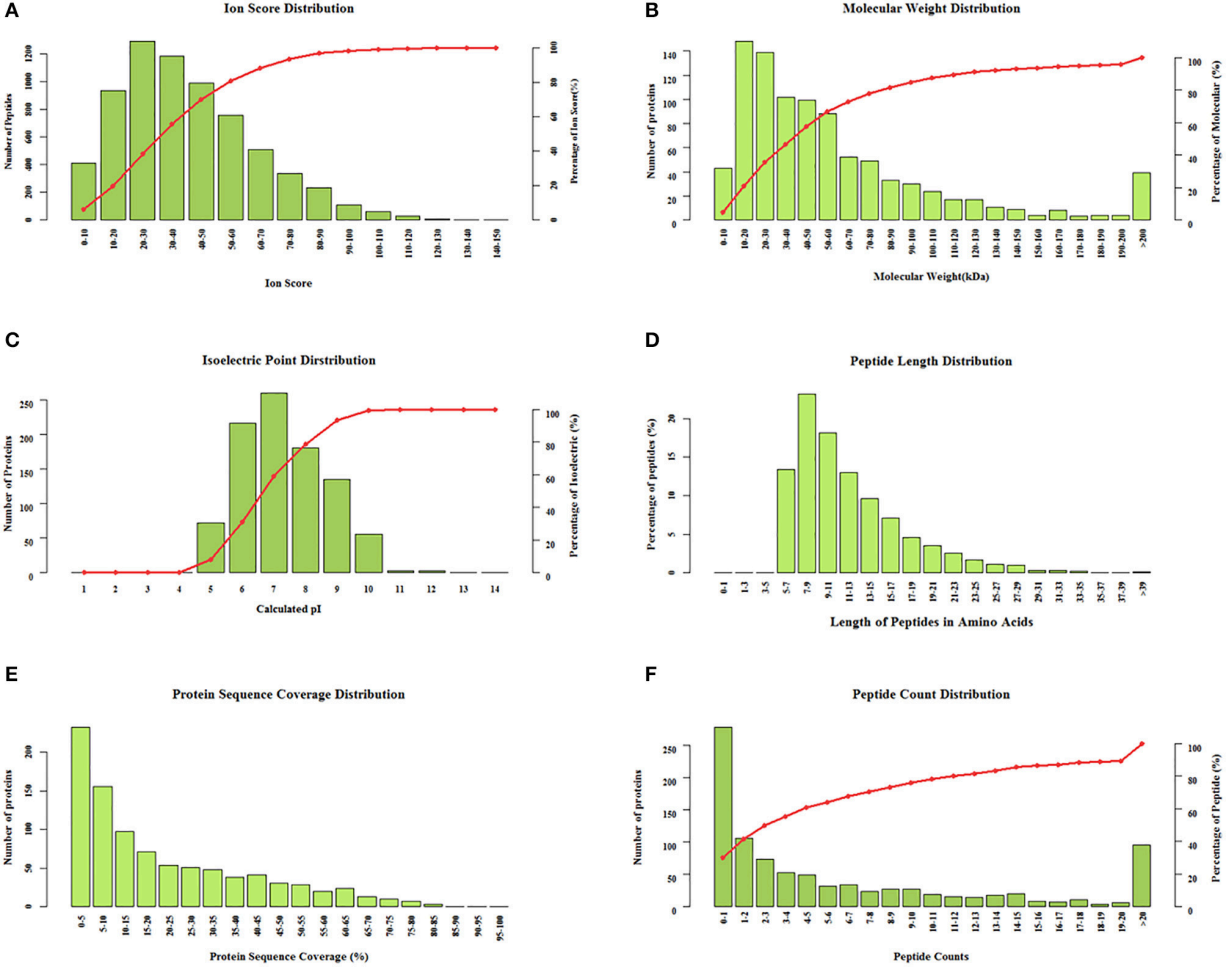
Identification and evaluation of the quantitative results from the tandem mass tag-based proteomics analysis. Protein distributions according to **(A)** ion score, **(B)** molecular weight, **(C)** isoelectric point, **(D)** peptide length, **(E)** protein sequence coverage, and **(F)** peptide count.

**Figure 5:**
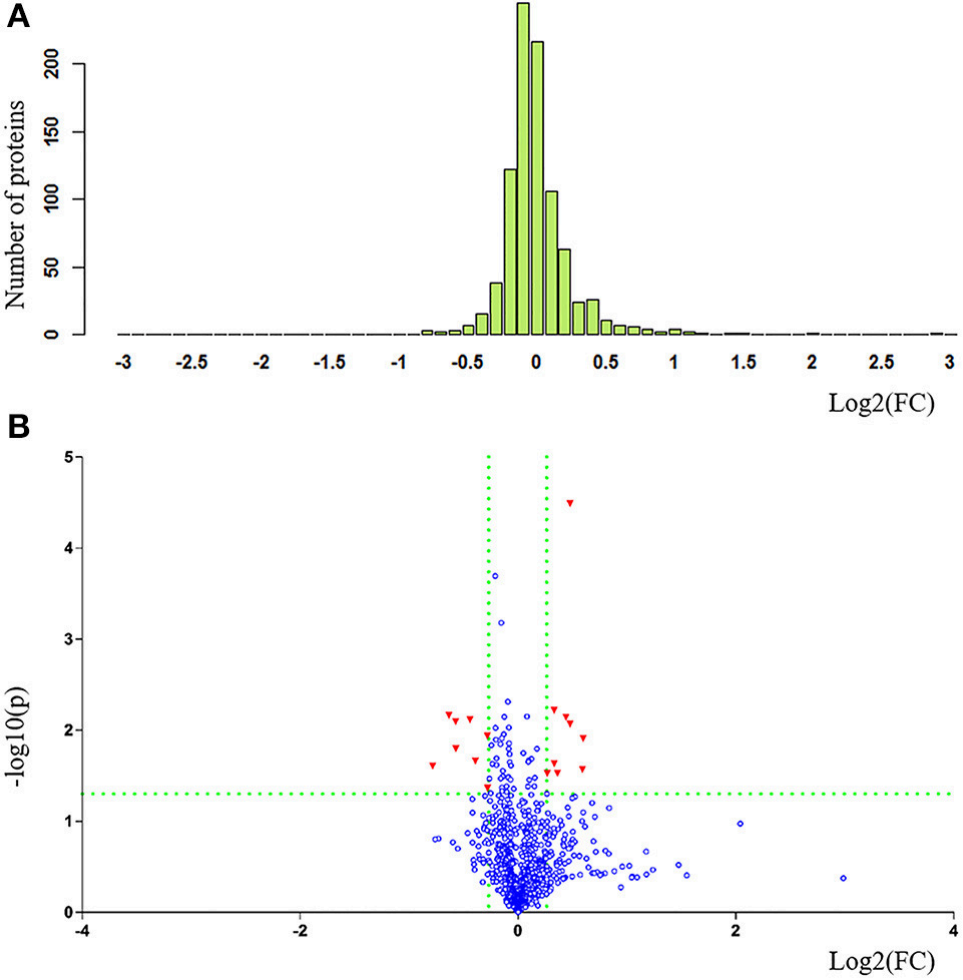
Statistical comparison and exhibition of proteins between patients with PDD and PD in proteomics analysis. **(A)** Protein ratio distribution and **(B)** volcano plot. FC value was calculated as the ratio of the average mass response (area) between the two groups (FC value = PDD/PD). *P*-value was calculated using Mann-Whitney *U*-test. PDD, Parkinson's disease-related depression; PD, Parkinson's disease; FC, fold change.

The clinical characteristics of patients included in the TMT-based proteomics analysis are shown in Supplementary Table 3 and were also comparable with the total patient group. Identification and evaluation of the quantitative proteomics results are shown in Figure 4. A total of 912 unique proteins were identified and protein ratio distributions between the two groups are shown in Figure 5. Only 17 differentially expressed proteins were selected for further analysis (Table 3).

**Table 3:** Key differentially expressed proteins identified by tandem mass tag-based proteomics analysis between patients with PD with and without depression.

**UniProt ID**	**Protein name (GeneName)**	**MW [kDa]**	**FC value^a^**	***p-*value^b^**
B3KY04	Potassium channel tetramerization domain containing 12 (KCTD12)	35.71	1.392	0.000
V9HW68	Epididymis luminal protein 214 (HEL-214)	51.68	1.258	0.006
Q68CJ9	Cyclic AMP-responsive element-binding protein 3-like protein 3 (CREB3L3)	49.05	1.357	0.007
E9PNH1	Neutral alpha-glucosidase AB (GANAB)	13.15	1.392	0.009
Q53HT9	Complement component 1, r subcomponent variant	80.19	1.514	0.012
P23471	Receptor-type tyrosine-protein phosphatase zeta (PTPRZ1)	254.43	1.259	0.023
A0A075B6R9	Protein IGKV2D-24 (IGKV2D-24)	13.07	1.506	0.027
A0A125U0U7	MS-C1 heavy chain variable region	13.09	1.286	0.030
Q04721	Neurogenic locus notch homolog protein 2 (NOTCH2)	265.23	1.204	0.030
P00338	L-lactate dehydrogenase A chain (LDHA)	36.67	0.644	0.007
U5YKD2	MHC class I antigen (HLA-A)	37.91	0.736	0.008
Q1HP67	Lipoprotein (LPA)	226.37	0.673	0.008
Q8NCM2	Potassium voltage-gated channel subfamily H member 5 (KCNH5)	111.81	0.822	0.012
E7EUT5	Glyceraldehyde-3-phosphate dehydrogenase (GAPDH)	27.85	0.673	0.016
Q5NV68	V4-1 protein (V4-1)	11.23	0.762	0.022
I3L252	Triokinase/FMN cyclase (TKFC)	22.94	0.581	0.025
D6RGG3	Collagen alpha-1(XII) chain (COL12A1)	333.00	0.824	0.043

a *FC value was calculated as the ratio of the average mass response (area) between the two groups (FC value = PDD/PD). Thus, FC values >1 indicate significantly higher levels in the PDD group relative to the PD group while FC values < 1 indicate significantly lower levels in the PDD group*.

b *Only proteins with FC values greater than ±1.2 and p values less than 0.05 were deemed statistically significant*.

*PD, Parkinson's disease; MW, molecular weight; FC, fold change; PDD, Parkinson's disease related depression*.

**Figure 6:**
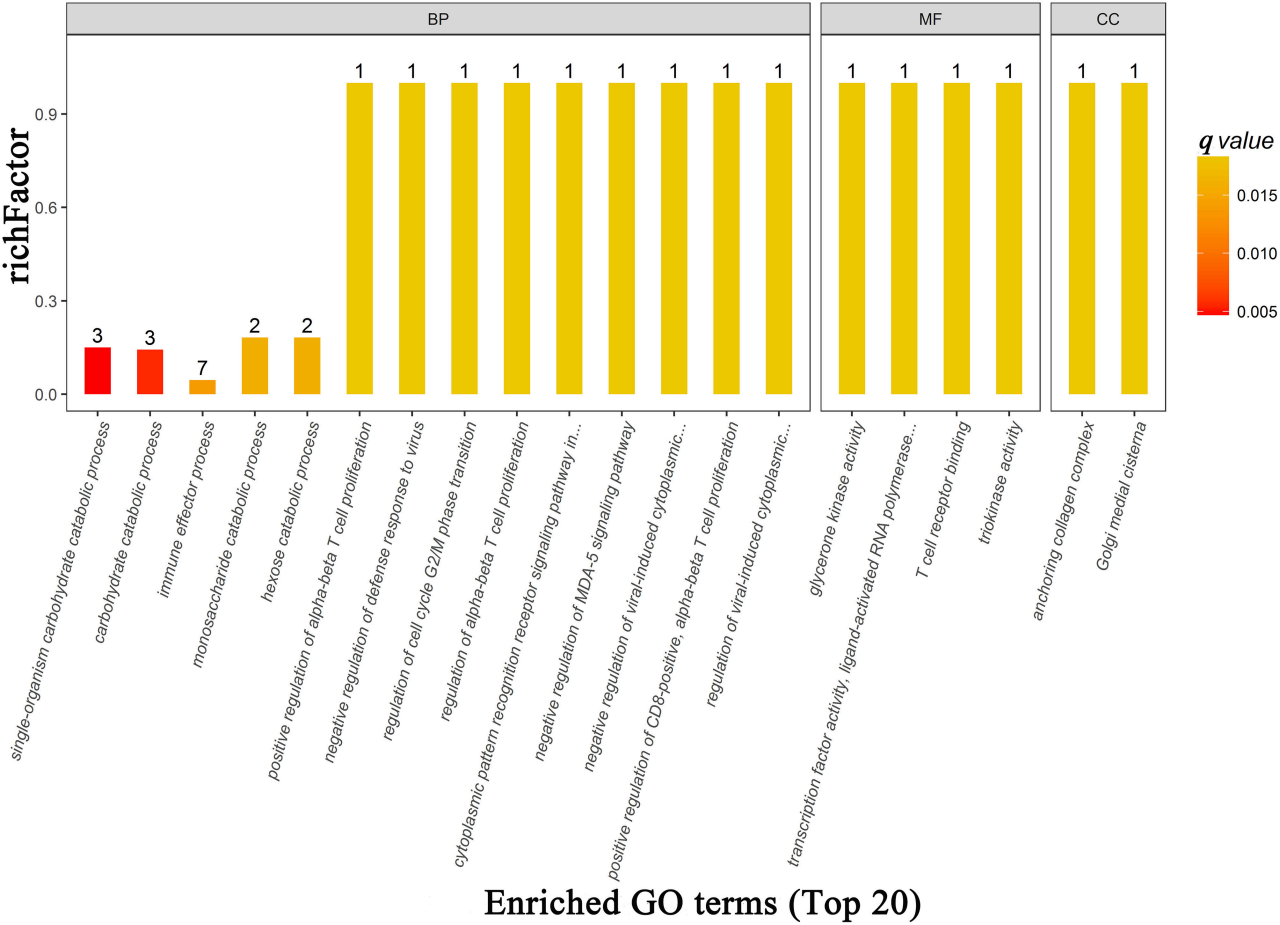
The top 20 most enriched GO terms based on proteomics analysis between patients with PDD and PD. BP, biological process; MF, molecular function; CC, cellular component; GO, gene ontology; PDD, Parkinson's disease-related depression; PD, Parkinson's disease.

There were 73, 14, 23 significant GO terms related to the differentially expressed proteins associated with biological process, cellular component, and molecular function, respectively. The top 20 GO terms of GO enrichment analysis are shown in Figure 6 and Supplementary Table 4. Several top ranking GO terms from biological processes were associated with glucose metabolism, including single-organism carbohydrate catabolic process, carbohydrate catabolic process, monosaccharide catabolic process, and hexose catabolic process. The top ranking enriched terms that related to molecular function were glycerone kinase activity, transcription factor activity, ligand-activated RNA polymerase II transcription factor binding, T cell receptor binding, and triokinase activity. The top ranking enriched terms that related to cellular components were anchoring collagen complex and Golgi medial cisterna.

Differentially expressed proteins were also mapped to KEGG pathways and enriched in 12 specific pathways (Figure 3B and Supplementary Table 5) with *q*-values < 0.05. There were six statistically overrepresented pathways involved in glucose metabolism, including glucagon signaling pathway, insulin secretion, glycolysis/gluconeogenesis, aldosterone synthesis and secretion, type I diabetes mellitus, and propanoate metabolism. All together, these findings further point to altered glucose metabolism in the PDD group.

### Integrated analysis

**Figure 7:**
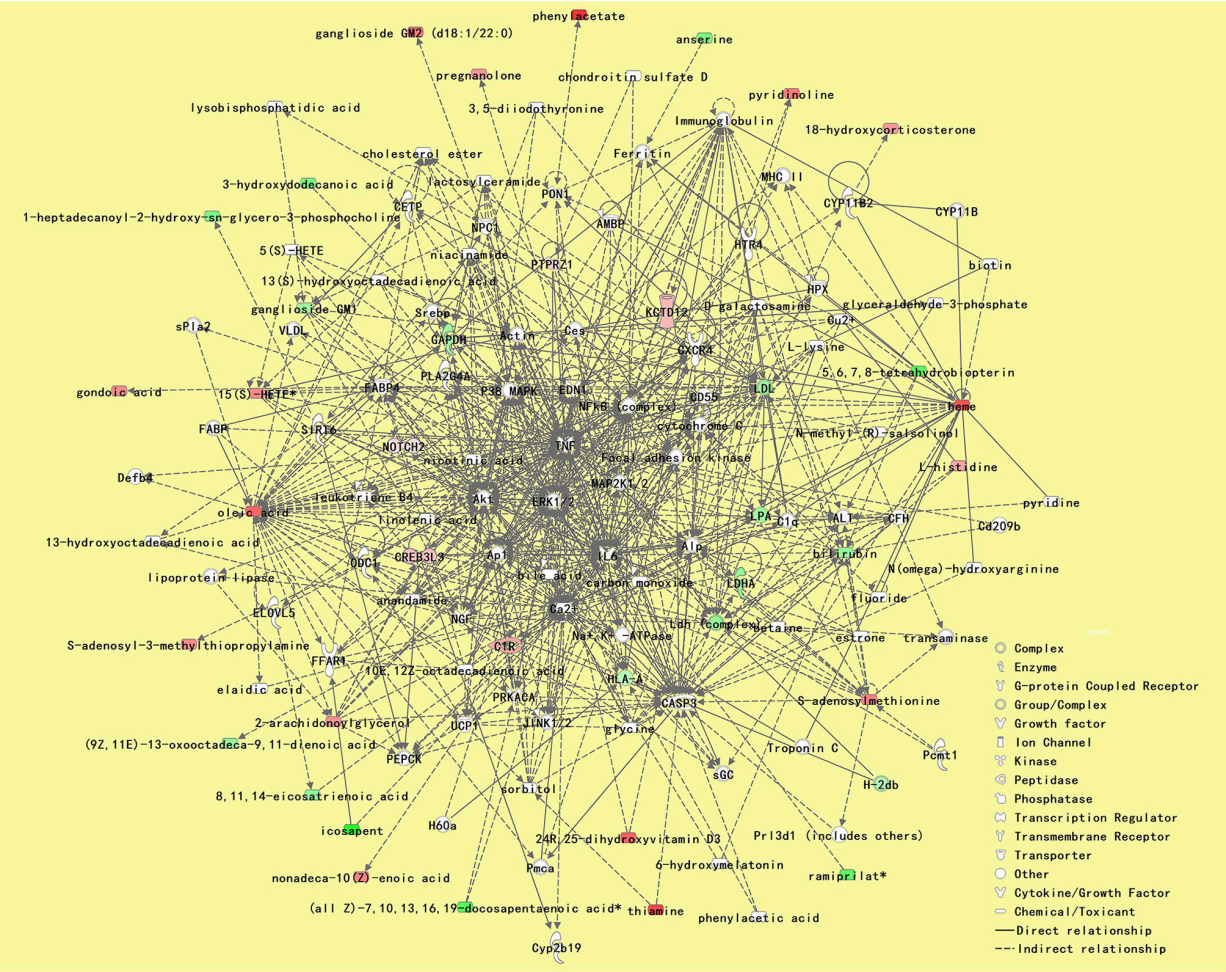
The most significant metabolite-protein integrated network between patients with PDD and PD. The network was “cellular compromise, lipid metabolism, and small molecule biochemistry” with a *p*score of 80, and a total of 27 metabolites and 9 proteins involved in the network. Upregulated metabolite symbols are in red, while green symbols indicate downregulated metabolites between patients with PDD and the other patients with PD. PDD, Parkinson's disease-related depression; PD, Parkinson's disease.

According to IPA analysis, these differentially expressed metabolites and proteins significantly relate to many molecular or cellular functions (Supplementary Table 6), further enriched in the following five categories: small molecule biochemistry, glucose metabolism, lipid metabolism, free radical scavenging, and cellular compromise (Figure 3C). The main metabolite-protein integrated network was “cellular compromise, lipid metabolism, and small molecule biochemistry” with a *p* score of 80 and a total of 27 metabolites and 9 proteins involved in the network (Figure 7).

### ELISA tests

**Figure 8:**
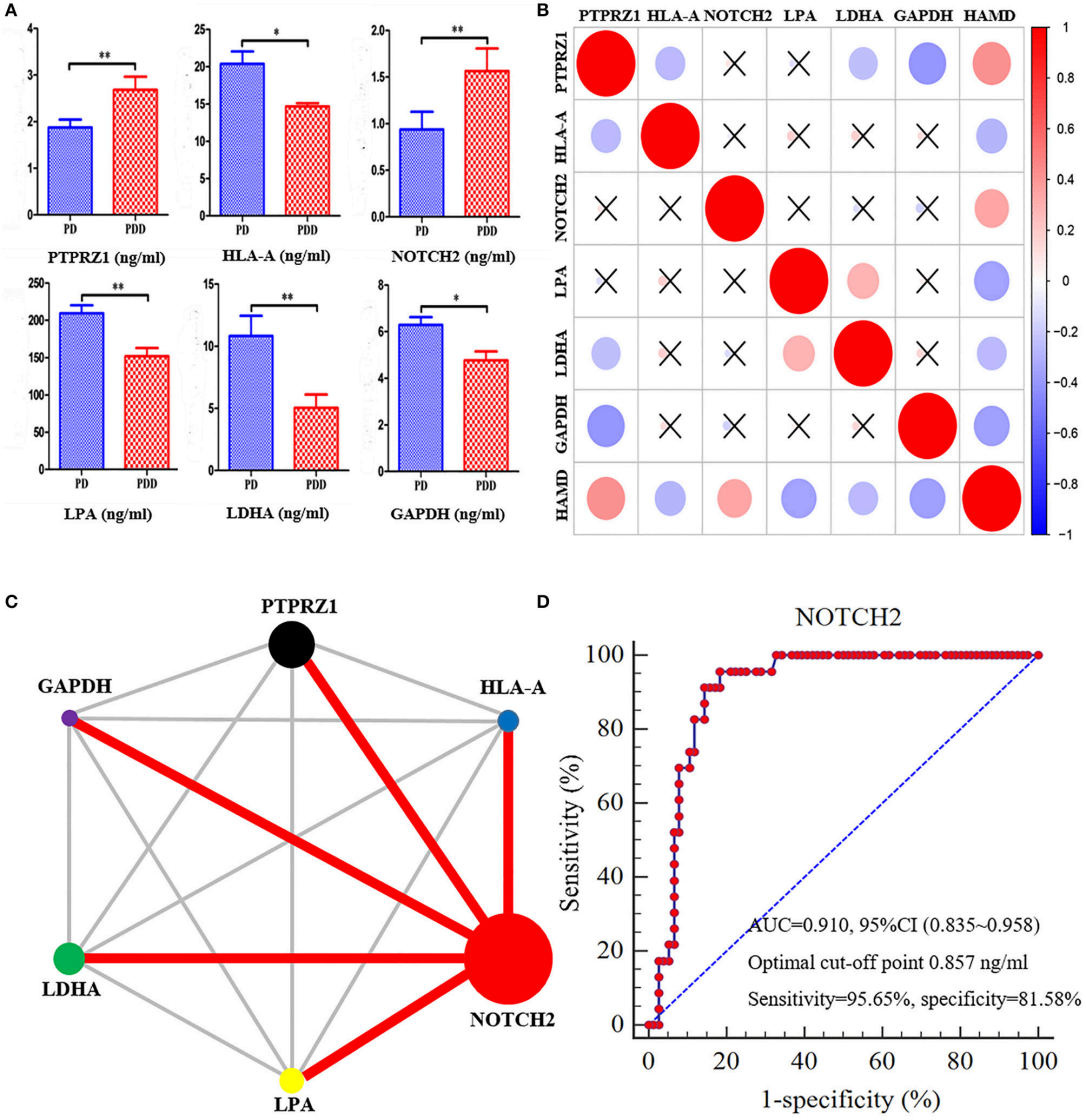
NOTCH2 may be a good potential biomarker for patients with PDD based on ROC curves. **(A)** Comparisons of the plasma levels of six proteins determined by ELISA tests between patients with PDD and the other patients with PD. The plasma levels of PTPRZ1 andNOTCH2 significantly increased in patients with PDD, whereas the plasma levels of HLA-A, LPA, LDHA, and GAPDH significantly decreased compared with the patients with PD. *p < 0.05 and **p < 0.01 compared with the patients with PD. **(B)** Correlation analyses between the plasma levels of six proteins and HAMD score in all patients with PD. The vertical bar on the right represents correlation coefficient value and “ × ” indicates no significant correlation can be found between them. The plot indicates positive associations of PTPRZ1 and NOTCH2, and negative associations of HLA-A, LPA, LDHA, and GAPDH, with HAMD scores. PTPRZ1 also exhibited negative associations with HLA-A, LDHA, and GAPDH, whereas LPA was positively associated with LDHA. **(C)** Comparison network of the diagnostic efficacies for patients with PDD. The circle area represents the AUC value of relevant proteins, and the red line indicates a statistically significant difference of the diagnostic efficacies between the two proteins. The AUC value of NOTCH2 was significantly higher than the others. **(D)** ROC curve of the plasma levels of NOTCH2 for detecting patients with PDD. PDD, Parkinson's disease-related depression; PD, Parkinson's disease; HAMD, Hamilton Depression Scale; ROC, receiver operating characteristic; AUC, area under the curve; CI, confidence interval.

According to the ELISA results (Table 4), the plasma levels of PTPRZ1 and NOTCH2 were significantly increased in patients with PDD, whereas the plasma levels of HLA-A, LPA, LDHA, and GAPDH were significantly decreased (Figure 8A). Correlation analyses revealed significant positive associations of PTPRZ1 and NOTCH2 and negative associations of HLA-A, LPA, LDHA, and GAPDH with HAMD scores (Figure 8B). PTPRZ1 also exhibited negative associations with HLA-A, LDHA, and GAPDH, whereas LPA was positively associated with LDHA. Receiver operating characteristic (ROC) curves were further analyzed and diagnostic efficacies were compared. Among them, the AUC value of NOTCH2 was significantly higher than the others. It suggested the plasma NOTCH2 level to be the best potential biomarker for patients with PDD (Figure 8C), with a sensitivity and specificity of 95.65 and 81.58%, respectively, and an optimal cut-off point of 0.91 ng/ml (Figure 8D).

**Table 4:** The plasma levels, Pearson correlation analyses with HAMD score, and ROC curves of the six proteins determined by ELISA tests in patients with PD with and without depression.

**Variable**	**Mean** ±**SEM (ng/ml)**			**Correlation analysis**		**ROC curve**			
	**PD (76)**	**PDD (23)**	***p*-value**	**r value**	***p* value**	**AUC (95%CI)**	**Cut-off point (ng/ml)**	**Sensitivity value (%)**	**Specificity value (%)**
PTPRZ1	1.88 ± 0.17	2.68 ± 0.28	0.000	0.417	0.000	0.758(0.662–0.838)	1.564	86.96	64.47
HLA-A	0.41 ± 0.03	0.29 ± 0.01	0.013	−0.272	0.006	0.672 (0.570–0.763)	18.738	100.0	39.47
NOTCH2	0.94 ± 0.19	1.56 ± 0.24	0.000	0.335	0.001	0.910 (0.835–0.958)	0.857	95.65	81.58
LPA	209.56 ± 10.76	151.90 ± 11.14	0.009	−0.354	0.000	0.681 (0.579–0.771)	216.827	95.65	44.74
LDHA	10.83 ± 1.62	5.05 ± 1.07	0.003	−0.251	0.012	0.707 (0.607–0.794)	4.542	78.26	63.16
GAPDH	6.29 ± 0.33	4.77 ± 0.39	0.028	−0.366	0.000	0.652 (0.550–0.745)	6.203	86.96	40.79

*HAMD, Hamilton Depression Scale; ROC, receiver operating characteristic; ELISA, enzyme-linked immune sorbent assay; PD, Parkinson's disease; SEM, standard error of the mean; ROC, receiver operator characteristics; PDD, Parkinson's disease related depression; AUC, area under the curve; CI, confidence interval; PTPRZ1, Receptor-type tyrosine-protein phosphatase zeta; HLA-A, Major histocompatibility complex class I antigen; NOTCH2, Neurogenic locus notch homolog protein 2; LPA, Lipoprotein; LDHA, L-lactate dehydrogenase A chain; GAPDH, Glyceraldehyde-3-phosphate dehydrogenase*.

## Discussion

Research bridging systems biology and clinical characteristics is useful to explore the underlying pathogenesis and to identify high quality clinical biomarkers (Mostafa et al., 2016). Here, we have performed an integrated metabolomics and proteomics analysis of PDD for the first time and have further identified potential blood biomarkers for depression in patients with PD.

No significant differences were found in most of the clinical characteristics, including age and gender, suggesting that our comparisons were reasonable. Hypertension is reportedly more prevalent in patients with PD (Hsu et al., 2015) and patients with depression (Yu et al., 2017), whereas patients with PDD in our research suffered less hypertension. According to a research from South Korea (Park et al., 2016), the incidence of hypertension is not significantly different between patients with PD with and without depression. This inconsistency is probably because the disease durations of patients with PD from South Korea were significantly shorter than those in our study (2.0 ± 1.0 vs. 6.0 ± 5.1 years). Orthostatic hypotension is reported to be associated with depressive symptoms in patients with PD, and that patients with PDD have attenuated cardiovagal dysfunction as observed during ambulation (Park et al., 2016). Decreased blood pressure in patients with PDD probably appears as the disease progresses. Serum lipid levels (triglyceride, Yu et al., 2017, LDL-C, Persons and Fiedorowicz, 2016, and high-density lipoprotein cholesterol, Ong et al., 2016) are also reportedly associated with depressive symptoms and suicidality (Oh and Kim, 2017). Here, we have demonstrated significantly lower serum LDL-C and Apo-B levels in patients with PDD for the first time, indicating that altered lipid metabolism is also associated with PDD. However, the incidence of hypercholesterolemia in patients with PDD exhibited no significant difference, probably because the altered lipid metabolism was well below that needed for a clinical diagnosis of hypercholesterolemia. Glucose metabolism is also reportedly associated with depressive symptoms in major depressive disorder (Koponen et al., 2015), post-stroke depression (Zhang et al., 2017b), and prenatal depressive symptoms (Huang et al., 2015). Patients with diabetes mellitus have a greater incidence of depressed mood compared to patients without diabetes mellitus (Levinger et al., 2012). Yet, the serum levels of blood glucose and HbA1C in our study showed no significant differences between patients with PDD and PD. In addition, the incidence of diabetes mellitus did not differ between patients with PD with and without depression (Icks et al., 2013). This is probably because the depressive systems are mainly correlated with impaired insulin sensitivity (Li et al., 2016) or low insulin secretion (Akbaraly et al., 2013), and the risk of depression gradually increases along with the deterioration of glucose metabolism (Chen et al., 2016).

Metabolomics analyses have already been performed in patients with PD (Trezzi et al., 2017) and patients with depression (Liu et al., 2015). Several metabolic pathways, including catecholamine metabolism, caffeine and xanthine pathways, ornithine pathway, and redox homoeostasis, are reportedly altered in patients with PD (Hatano et al., 2016), while depressive symptoms are associated with lipid metabolism, carnitine metabolism, amino acid metabolism, and bile acid metabolism (Liu et al., 2015). Nevertheless, this metabolomics analysis was the first to be performed in patients with PDD compared with patients with PD and significant metabolite changes were identified. Most of the differentially expressed metabolites were categorized into lipids and lipid-like molecules. Four carbohydrates had also changed. The involvement of lipid metabolism in PDD is consistent with the above clinical characteristics (lower LDL-C and Apo-B level) and can further be confirmed by the metabolic pathway analysis. The most significant and important pathway was “alpha linolenic acid and linoleic acid metabolism,” participating in the metabolism of essential polyunsaturated fatty acids. This metabolic pathway is reportedly associated with the occurrence (Lucas et al., 2011) and treatment (Kanno et al., 2015) of depression, and the weak positive association between the intake of polyunsaturated fatty acid and PD risk needs further investigation (Dong et al., 2014). The second most significantly altered metabolic pathway was “incretin synthesis, secretion, and inactivation,” which is related to glucose metabolism. The decreased secretion and sensitivity level of insulin, another common hypoglycemic hormone, appears to be a risk factor for depression (Akbaraly et al., 2013; Li et al., 2016). However, this is the first report of the synthesis, secretion, and inactivation of incretin involved in patients with PDD, and the relationship between incretin and depressive symptoms in patients with PD needs further clarification. The last significant pathway was “signal transduction,” by which a chemical or physical signal is transmitted through a cell, ultimately resulting in a cellular response (Hynes et al., 2013). The realization of altered lipid and glucose metabolism is also through such a process.

A previous serum proteomics analysis revealed that oxidative stress, mitochondrial dysfunction, abnormal protein aggregation, and inflammation may be associated with PD (Zhang et al., 2012), while patients with depression may have altered lipid metabolism and immunoregulation (Xu et al., 2012). In this TMT-based proteomics analysis, 17 differentially expressed proteins were identified for further analysis. According to the GO enrichment analysis, four of the top five ranking GO terms from biological processes were associated with glucose metabolism, including single-organism carbohydrate catabolic process, carbohydrate catabolic process, monosaccharide catabolic process, and hexose catabolic process. Enriched KEGG pathway analysis was also performed and 12 statistically overrepresented pathways were mapped, among which six pathways were involved in glucose metabolism, including glucagon signaling pathway, insulin secretion, glycolysis/gluconeogenesis, aldosterone synthesis and secretion, type I diabetes mellitus, and propanoate metabolism. Those findings at the protein level firmly support the results of the metabolomics analysis. Although the involvement of lipid metabolism was not confirmed at the protein level by pathway analysis, lysophosphatidic acid (LPA), a common protein related to lipid metabolism was significantly increased in patients with PDD based on proteomics analysis. In other words, both glucose and lipid participate in the energy metabolism of a biological system. It is just the alteration of glucose metabolism at the protein level that induces disturbance of lipid and glucose metabolism at the metabolite level.

Metabolic pathways involve both metabolites and proteins, and integrated analysis of both metabolomics and proteomics data will thus help us interpret the above inconsistence (Zhang et al., 2017a). When incorporating all the differentially expressed metabolites and proteins onto indole-3-propionic acid (IPA), those differentially expressed molecules predominantly participated in small molecule biochemistry, glucose metabolism, lipid metabolism, free radical scavenging, and cellular compromise. Small molecule biochemistry is the metabolic processes of many small molecules, including glucose and lipid metabolism, consistent with the above results of the clinical characteristics, metabolomics, and proteomics. Oxidative stress is one of the mechanisms of cellular damage and can also induce depressive symptoms according to previous research (Pan et al., 2014; Bouvier et al., 2016). The enriched category of free radical scavenging in this study includes the production and activation of reactive oxygen species, while cellular compromise includes cytotoxicity and cellular injury (Supplementary Table 6). Those results demonstrate that altered lipid and glucose metabolism induces depressive symptoms in patients with PD, possibly also through oxidative stress and the resulting cellular injury. The most significant integrated network in this study was “cellular compromise, lipid metabolism, and small molecule biochemistry,” with a total of 27 metabolites and 9 proteins involved. Those molecules in the network were recognized as the most relevant ones with depressive systems in patients with PD (Dong et al., 2017b). We further performed ELISA tests for six differentially expressed proteins to identify some clinical biomarkers as an integral part of clinical researches (Mostafa et al., 2016).

The expression differences of those proteins were confirmed. Correlation analyses revealed significant positive associations of PTPRZ1 and NOTCH2 and negative associations of HLA-A, LPA, LDHA, and GAPDH, with HAMD scores. Significant correlations were also found between these proteins. PTPRZ1 exhibited negative associations with HLA-A, LDHA, and GAPDH, whereas LPA was positively associated with LDHA. For these proteins ROC curves were performed, and the comparison of diagnostic efficacies identified the plasma NOTCH2 level to be the best potential blood biomarker for PDD (with a significantly higher AUC value than the others). NOTCH2 can be expressed in various cell types from the hematolymphoid compartment and has specific roles in differentiation and function of many immune cells (Sakata-Yanagimoto and Chiba, 2012). Genetic variants of NOTCH2 reportedly can also increase susceptibility to diabetes mellitus (Pan et al., 2013). Hence, the increased plasma NOTCH2 level in patients with PD induces depressive symptoms probably also through glucose metabolism.

There were several limitations to this study. First, the number of patients included was relatively small, and all the patients were from a Chinese Han population. The results require further confirmation, especially in another ethnicity. Second, we have only determined six differentially expressed proteins according to the metabolite-protein integrated network. More antibodies and ELISA kits should be developed to validate the alteration of the other proteins. We hope to continue the research about PDD and overcome these limitations in the future.

## Conclusions

The integrated metabolomics and proteomics analysis from this study reveals that depressive symptoms in patients with PD are predominantly associated with lipid and glucose metabolism. Plasma NOTCH2 level may be a good blood biomarker for detecting patients of PD with depression.

## Data availability statements

The raw data supporting the conclusions of this manuscript will be made available by the authors, without undue reservation, to any qualified researcher.

## Author contributions

M-XD and Y-DW designed this study. M-XD, XF, X-MX, YL, S-YJ, and LH assessed clinical data. M-XD, BL, and WC performed experiments. M-XD wrote the manuscript. Y-DW revised it.

### Conflict of interest statement

WC was employed by Shanghai Applied Protein Technology Co., Ltd. The remaining authors declare that the research was conducted in the absence of any commercial or financial relationships that could be construed as a potential conflict of interest.

## References

[B1] Aarsland D. Zaccai J. Brayne C. (2005). A systematic review of prevalence studies of dementia in Parkinson's disease. Mov. Disord 20, 1255–1263. 10.1002/mds.20527 16041803

[B2] Adams-Carr K. L. Bestwick J. P. Shribman S. Lees A. Schrag A. Noyce A. J. (2016). Constipation preceding Parkinson's disease: a systematic review and meta-analysis. J. Neurol. Neurosurg. Psychiatry 87, 710–716. 10.1136/jnnp-2015-311680 26345189

[B3] Akbaraly T. N. Kumari M. Head J. Ritchie K. Ancelin M. L. Tabak A. G.. (2013). Glycemia, insulin resistance, insulin secretion, and risk of depressive symptoms in middle age. Diab. Care 36, 928–934. 10.2337/dc12-0239 23230097 PMC3609527

[B4] Altelaar A. F. Munoz J. Heck A. J. (2013). Next-generation proteomics: towards an integrative view of proteome dynamics. Nat. Rev. Genet. 14, 35–48. 10.1038/nrg3356 23207911

[B5] Berardelli A. Wenning G. K. Antonini A. Berg D. Bloem B. R. Bonifati V.. (2013). EFNS/MDS-ES/ENS [corrected] recommendations for the diagnosis of Parkinson's disease. Eur. J. Neurol. 20, 16–34. 10.1111/ene.12022 23279440

[B6] Berger B. Peng J. Singh M. (2013). Computational solutions for omics data. Nat. Rev. Genet. 14, 333–346. 10.1038/nrg3433 23594911 PMC3966295

[B7] Bouvier E. Brouillard F. Molet J. Claverie D. Cabungcal J. H. Cresto N. (2016). Nrf2-dependent persistent oxidative stress results in stress-induced vulnerability to depression. Mol. Psychiatry 22, 1701–1713. 10.1038/mp.2016.144 27646262

[B8] Broen M. P. Narayen N. E. Kuijf M. L. Dissanayaka N. N. Leentjens A. F. (2016). Prevalence of anxiety in Parkinson's disease: a systematic review and meta-analysis. Mov. Disord. 31, 1125–1133. 10.1002/mds.26643 27125963

[B9] Campbell K. Xia J. Nielsen J. (2017). The impact of systems biology on bioprocessing. Trends Biotechnol. 35, 1156–1168. 10.1016/j.tibtech.2017.08.011 28987922

[B10] Chen S. Zhang Q. Dai G. Hu J. Zhu C. Su L.. (2016). Association of depression with pre-diabetes, undiagnosed diabetes, and previously diagnosed diabetes: a meta-analysis. Endocrine 53, 35–46. 10.1007/s12020-016-0869-x 26832340

[B11] Dayon L. Nunez Galindo A. Corthesy J. Cominetti O. Kussmann M. (2014). Comprehensive and Scalable Highly Automated MS-Based Proteomic Workflow for Clinical Biomarker Discovery in Human Plasma. J. Proteome Res. 13, 3837–3845. 10.1021/pr500635f 25058407

[B12] Dissanayaka N. N. Sellbach A. Matheson S. Marsh R. Silburn P. A. O'sullivan J. D.. (2007). Validity of Hamilton Depression Inventory in Parkinson's disease. Mov. Disord. 22, 399–403. 10.1002/mds.21309 17230471

[B13] Dong J. Beard J. D. Umbach D. M. Park Y. Huang X. Blair A.. (2014). Dietary fat intake and risk for Parkinson's disease. Mov. Disord. 29, 1623–1630. 10.1002/mds.26032 25186946 PMC4216604

[B14] Dong M. X. Hu L. Huang Y. J. Xu X. M. Liu Y. Wei Y. D. (2017a). Cerebrovascular risk factors for patients with cerebral watershed infarction: a case-control study based on computed tomography angiography in a population from Southwest China. Medicine 96:e7505. 10.1097/MD.0000000000007505 28700499 PMC5515771

[B15] Dong M. X. Hu Q. C. Shen P. Pan J. X. Wei Y. D. Liu Y. Y.. (2016). Recombinant Tissue Plasminogen Activator Induces Neurological Side Effects Independent on Thrombolysis in Mechanical Animal Models of Focal Cerebral Infarction: a Systematic Review and Meta-Analysis. PLoS ONE 11:e0158848. 10.1371/journal.pone.0158848 27387385 PMC4936748

[B16] Dong M. X. Li C. M. Shen P. Hu Q. C. Wei Y. D. Ren Y. F.. (2017b). Recombinant tissue plasminogen activator induces long-term anxiety-like behaviors via the ERK1/2-GAD1-GABA cascade in the hippocampus of a rat model. Neuropharmacology 128, 119–131. 10.1016/j.neuropharm.2017.09.039 28986280

[B17] Dong M. X. Xu X. M. Hu L. Liu Y. Huang Y. J. Wei Y. D. (2017c). Serum Butyrylcholinesterase Activity: A Biomarker for Parkinson's Disease and Related Dementia. Biomed. Res. Int. 2017:1524107. 10.1155/2017/1524107 28840123 PMC5559914

[B18] Goodarzi Z. Mrklas K. J. Roberts D. J. Jette N. Pringsheim T. Holroyd-Leduc J. (2016). Detecting depression in Parkinson disease: a systematic review and meta-analysis. Neurology 87, 426–437. 10.1212/WNL.0000000000002898 27358339 PMC4977107

[B19] Gustafsson H. Nordstrom A. Nordstrom P. (2015). Depression and subsequent risk of Parkinson disease: a nationwide cohort study. Neurology 84, 2422–2429. 10.1212/WNL.0000000000001684 25995056 PMC4478031

[B20] Hatano T. Saiki S. Okuzumi A. Mohney R. P. Hattori N. (2016). Identification of novel biomarkers for Parkinson's disease by metabolomic technologies. J. Neurol Neurosurg. Psychiatry 87, 295–301. 10.1136/jnnp-2014-309676 25795009

[B21] Hochberg Y. Benjamini Y. (1990). More powerful procedures for multiple significance testing. Stat. Med. 9, 811–818. 10.1002/sim.4780090710 2218183

[B22] Hsu Y. T. Liao C. C. Chang S. N. Yang Y. W. Tsai C. H. Chen T. L.. (2015). Increased Risk of Depression in Patients with Parkinson Disease: a Nationwide Cohort Study. Am. J. Geriatr. Psychiatry 23, 934–940. 10.1016/j.jagp.2014.10.011 25529799

[B23] Hu L. Dong M. X. Zhao H. Xu G. H. Qin X. Y. (2016). Fibulin-5: a novel biomarker for evaluating severity and predicting prognosis in patients with acute intracerebral haemorrhage. Eur. J. Neurol. 23, 1195–1201. 10.1111/ene.13013 27106135

[B24] Huang T. Rifas-Shiman S. L. Ertel K. A. Rich-Edwards J. Kleinman K. Gillman M. W.. (2015). Pregnancy hyperglycaemia and risk of prenatal and postpartum depressive symptoms. Paediatr. Perinat. Epidemiol. 29, 281–289. 10.1111/ppe.12199 26058318 PMC4642439

[B25] Hynes N. Ingham P. Lim W. Marshall C. Massagu,é J. Pawson T. (2013). Signalling change: signal transduction through the decades. Nat. Rev. Mol. Cell Biol. 14, 393–398. 10.1038/nrm3581 23636498

[B26] Icks A. Albers B. Haastert B. Pechlivanis S. Bokhof B. Slomiany U. (2013). Diabetes incidence does not differ between subjects with and without high depressive symptoms−5-year follow-up results of the Heinz Nixdorf Recall Study. Diabet. Med. 30, 65–69. 10.1111/j.1464-5491.2012.03724.x 22672118

[B27] Kalia L. Lang A. (2015). Parkinson's disease. Lancet 386, 896–912. 10.1016/S0140-6736(14)61393-3 25904081

[B28] Kamburov A. Cavill R. Ebbels T. M. Herwig R. Keun H. C. (2011). Integrated pathway-level analysis of transcriptomics and metabolomics data with IMPaLA. Bioinformatics 27, 2917–2918. 10.1093/bioinformatics/btr499 21893519

[B29] Kanno T. Tanaka A. Nishizaki T. (2015). Linoleic acid derivative DCP-LA ameliorates stress-induced depression-related behavior by promoting cell surface 5-HT1A receptor translocation, stimulating serotonin release, and inactivating GSK-3beta. Mol. Neurobiol. 51, 523–532. 10.1007/s12035-014-8718-5 24788685

[B30] Koponen H. Kautiainen H. Leppanen E. Mantyselka P. Vanhala M. (2015). Association between suicidal behaviour and impaired glucose metabolism in depressive disorders. BMC Psychiatry 15:163. 10.1186/s12888-015-0567-x 26199013 PMC4509469

[B31] Kowal S. L. Dall T. M. Chakrabarti R. Storm M. V. Jain A. (2013). The current and projected economic burden of Parkinson's disease in the United States. Mov. Disord. 28, 311–318. 10.1002/mds.25292 23436720

[B32] Levinger I. Selig S. Jerums G. Stewart A. Gaskin C. J. Hare D. L. (2012). Depressed mood, glycaemic control and functional capacity in overweight/obese men with and without type 2 diabetes. Diabetol. Metab. Syndr. 4:46. 10.1186/1758-5996-4-46 23171832 PMC3520815

[B33] Li L. Shelton R. C. Chassan R. A. Hammond J. C. Gower B. A. Garvey T. W. (2016). Impact of major depressive disorder on prediabetes by impairing insulin sensitivity. J. Diab. Metab. 7:664. 10.4172/2155-6156.1000664 27274905 PMC4892179

[B34] Liu X. Zheng P. Zhao X. Zhang Y. Hu C. Li J.. (2015). Discovery and validation of plasma biomarkers for major depressive disorder classification based on liquid chromatography-mass spectrometry. J. Proteome Res. 14, 2322–2330. 10.1021/acs.jproteome.5b00144 25784130

[B35] Lucas M. Mirzaei F. O'reilly E. J. Pan A. Willett W. C. Kawachi I.. (2011). Dietary intake of n-3 and n-6 fatty acids and the risk of clinical depression in women: a 10-y prospective follow-up study. Am. J. Clin. Nutr. 93, 1337–1343. 10.3945/ajcn.111.011817 21471279 PMC3095504

[B36] Macleod A. D. Taylor K. S. Counsell C. E. (2014). Mortality in Parkinson's disease: a systematic review and meta-analysis. Mov. Disord. 29, 1615–1622. 10.1002/mds.25898 24821648

[B37] Marsh L. (2013). Depression and Parkinson's disease: current knowledge. Curr. Neurol. Neurosci. Rep. 13:409. 10.1007/s11910-013-0409-5 24190780 PMC4878671

[B38] Mostafa I. Zhu N. Yoo M. J. Balmant K. M. Misra B. B. Dufresne C.. (2016). New nodes and edges in the glucosinolate molecular network revealed by proteomics and metabolomics of Arabidopsis myb28/29 and cyp79B2/B3 glucosinolate mutants. J. Proteomics 138, 1–19. 10.1016/j.jprot.2016.02.012 26915584

[B39] Oh J. Kim T. S. (2017). Serum lipid levels in depression and suicidality: the Korea National Health and Nutrition Examination Survey (KNHANES) 2014. J. Affect Disord. 213, 51–58. 10.1016/j.jad.2017.02.002 28189965

[B40] Ong K. L. Morris M. J. Mcclelland R. L. Maniam J. Allison M. A. Rye K. A. (2016). Lipids, lipoprotein distribution and depressive symptoms: the Multi-Ethnic Study of Atherosclerosis. Trans. Psychiatry 6:e962. 10.1038/tp.2016.232 27898070 PMC5290355

[B41] Pan J. Liu H. Zhou J. Liu Z. Yang Y. Peng Y.. (2014). Ipsilateral hippocampal proteomics reveals mitochondrial antioxidative stress impairment in cortical-lesioned chronic mild stressed rats. Curr. Mol. Med. 14, 1186–1196. 10.2174/1566524014666141021143333 25336330

[B42] Pan W. C. Kile M. L. Seow W. J. Lin X. Quamruzzaman Q. Rahman M.. (2013). Genetic susceptible locus in NOTCH2 interacts with arsenic in drinking water on risk of type 2 diabetes. PLoS ONE 8:e70792. 10.1371/journal.pone.0070792 23967108 PMC3743824

[B43] Park H. E. Kim J. S. Oh Y. S. Park I. S. Park J. W. Song I. U.. (2016). Autonomic nervous system dysfunction in patients with Parkinson disease having depression. J. Geriatr. Psychiatry Neurol. 29, 11–17. 10.1177/0891988715598234 26232405

[B44] Persons J. E. Fiedorowicz J. G. (2016). Depression and serum low-density lipoprotein: a systematic review and meta-analysis. J. Affect. Disord 206, 55–67. 10.1016/j.jad.2016.07.033 27466743 PMC6201299

[B45] Ponsen M. Stoffers D. Twisk J. Wolters E. Berendse H. (2009). Hyposmia and executive dysfunction as predictors of future Parkinson's disease: a prospective study. Mov. Disord. 24, 1060–1065. 10.1002/mds.22534 19353591

[B46] Postuma R. B. Berg D. Stern M. Poewe W. Olanow C. W. Oertel W.. (2015). MDS clinical diagnostic criteria for Parkinson's disease. Mov. Disord 30, 1591–1601. 10.1002/mds.26424 26474316

[B47] Pringsheim T. Jette N. Frolkis A. Steeves T. D. (2014). The prevalence of Parkinson's disease: a systematic review and meta-analysis. Mov. Disord 29, 1583–1590. 10.1002/mds.25945 24976103

[B48] Reijnders J. S. Ehrt U. Weber W. E. Aarsland D. Leentjens A. F. (2008). A systematic review of prevalence studies of depression in Parkinson's disease. Mov. Disord. 23, 183–189:quiz 313. 10.1002/mds.21803 17987654

[B49] Sakata-Yanagimoto M. Chiba S. (2012). Notch2 and immune function. Curr. Top. Microbiol. Immunol. 360, 151–161. 10.1007/82_2012_235 22695918

[B50] Sharma S. Moon C. S. Khogali A. Haidous A. Chabenne A. Ojo C.. (2013). Biomarkers in Parkinson's disease (recent update). Neurochem. Int. 63, 201–229. 10.1016/j.neuint.2013.06.005 23791710

[B51] Shen P. Hu Q. Dong M. Bai S. Liang Z. Chen Z.. (2017). Venlafaxine exerts antidepressant effects possibly by activating MAPK-ERK1/2 and P13K-AKT pathways in the hippocampus. Behav. Brain Res. 335, 63–70. 10.1016/j.bbr.2017.08.011 28797602

[B52] Sogawa K. Takano S. Iida F. Satoh M. Tsuchida S. Kawashima Y.. (2016). Identification of a novel serum biomarker for pancreatic cancer, C4b-binding protein α-chain (C4BPA) by quantitative proteomic analysis using tandem mass tags. Br. J. Cancer 115, 949–956. 10.1038/bjc.2016.295 27657339 PMC5061912

[B53] Starkstein S. E. Merello M. Jorge R. Brockman S. Bruce D. Petracca G.. (2008). A validation study of depressive syndromes in Parkinson's disease. Mov. Disord 23, 538–546. 10.1002/mds.21866 18074376

[B54] Trezzi J. P. Galozzi S. Jaeger C. Barkovits K. Brockmann K. Maetzler W.. (2017). Distinct metabolomic signature in cerebrospinal fluid in early parkinson's disease. Mov. Disord 32, 1401–1408. 10.1002/mds.27132 28843022

[B55] Wishart D. S. (2016). Emerging applications of metabolomics in drug discovery and precision medicine. Nat. Rev. Drug Discov. 15, 473–484. 10.1038/nrd.2016.32 26965202

[B56] Xu H. B. Zhang R. F. Luo D. Zhou Y. Wang Y. Fang L.. (2012). Comparative proteomic analysis of plasma from major depressive patients: identification of proteins associated with lipid metabolism and immunoregulation. Int. J. Neuropsychopharmacol. 15, 1413–1425. 10.1017/S1461145712000302 22717272

[B57] Yu S. Yang H. Guo X. Zheng L. Sun Y. (2017). Metabolic syndrome and depressive symptoms among rural Northeast general population in China. BMC Public Health 17:43. 10.1186/s12889-016-3913-0 28061774 PMC5219740

[B58] Zhang A. Zhou X. Zhao H. Zou S. Ma C. W. Liu Q.. (2017a). Metabolomics and proteomics technologies to explore the herbal preparation affecting metabolic disorders using high resolution mass spectrometry. Mol. Biosyst. 13, 320–329. 10.1039/c6mb00677a 28045158

[B59] Zhang X. Yin X. Yu H. Liu X. Yang F. Yao J.. (2012). Quantitative proteomic analysis of serum proteins in patients with Parkinson's disease using an isobaric tag for relative and absolute quantification labeling, two-dimensional liquid chromatography, and tandem mass spectrometry. Analyst 137, 490–495. 10.1039/C1AN15551B 22108571

[B60] Zhang Y. He J. R. Liang H. B. Lu W. J. Yang G. Y. Liu J. R.. (2017b). Diabetes mellitus is associated with late-onset post-stroke depression. J. Affect. Disord. 221, 222–226. 10.1016/j.jad.2017.06.045 28654846

[B61] Zhao P. Li J. Li Y. Tian Y. Yang L. Li S. (2017). Integrating Transcriptomics, Proteomics, and Metabolomics Profiling with System Pharmacology for the Delineation of Long-Term Therapeutic Mechanisms of Bufei Jianpi Formula in Treating COPD. Biomed. Res. Int. 2017:7091087. 10.1155/2017/7091087 28424787 PMC5382313

[B62] Zheng P. Zeng B. Zhou C. Liu M. Fang Z. Xu X.. (2016). Gut microbiome remodeling induces depressive-like behaviors through a pathway mediated by the host's metabolism. Mol. Psychiatry 21, 786–796. 10.1038/mp.2016.44 27067014

