# Supplementary material for: Integrated Analysis Reveals Altered Lipid and Glucose Metabolism and Identifies NOTCH2 as a Biomarker for Parkinson's Disease Related Depression
Source: Front Mol Neurosci. 2018 Aug 31;11:257. doi: 10.3389/fnmol.2018.00257 (PMC6127515; doi:10.3389/fnmol.2018.00257)
Supplement: Supplementary file 1 [file Data_Sheet_1.PDF]

Supplementary materials

Supplementary eTable 1. Clinical characteristics of PD patients with and without depression included in the liquid chromatography-mass spectrometry-based metabolomics analysis.

Variable (SEM/%)	PD (40)	PDD (23)	*p* value	Variable (SEM/%)	PD (40)	PDD (23)	*p* value
Age (year)	66.78±1.31	66.48±2.16	0.901	RBG (mmol/L)	6.95±0.28	7.28±0.62	0.575
Gender, Male (%)	23 (57.5%)	10 (43.5%)	0.283	HbA1C (%)	6.06±0.13%	7.09±0.85%	0.270
Smoking history (%)	11 (27.5%)	2 (8.7%)	0.146	TC (mmol/L)	4.55±0.12	3.70±0.29	0.003
Alcohol consumption (%)	4 (10.0%)	1 (4.3%)	0.644	TG (mmol/L)	1.36±0.10	1.12±0.16	0.202
Hypertension (%)	16 (40.0%)	4 (17.4%)	0.063	HDL-C (mmol/L)	1.34±0.05	1.49±0.07	0.086
Diabetes mellitus (%)	6 (15.0%)	4 (17.4%)	1.000	LDL-C (mmol/L)	2.96±0.12	2.37±0.15	0.004
Hypercholesterolemia (%)	12 (30.0%)	2 (5.1%)	0.050	Apo-A1 (g/L)	1.33±0.03	1.38±0.04	0.413
CHD (%)	5 (12.5%)	4 (17.4%)	0.873	Apo-B (g/L)	0.92±0.03	0.74±0.04	0.001
Disease duration (year)	4.78±0.65	7.35±0.93	0.023	HAMD score	8.08±0.80	21.48±0.57	0.000
BMI (kg/m^2)	23.81±0.57	22.25±0.67	0.089				

PD, Parkinson's disease; SEM, standard error of the mean; PDD, Parkinson's disease related depression; RBG, random blood glucose; HbA1C, hemoglobin A1C; TC, total cholesterol; TG, triglyceride; HDL-C, high-density lipoprotein cholesterol; LDL-C, low-density lipoprotein cholesterol; Apo-A1, apolipoprotein A1; CHD, coronary heart disease; Apo-B, apolipoprotein B; HAMD, Hamilton Depression Scale; BMI, body mass index.

Supplementary eTable 2. The top ten most significant metabolic pathways determined by liquid chromatography-mass spectrometry-based metabolomics analysis obtained from Integrated Molecular Pathway Level Analysis (impala.molgen.mpg.de).

Pathway name	Pathway source	Number of overlapping metabolites	Number of total pathway metabolites	Pathway impact	*p* value	*q* value
Alpha linolenic acid and linoleic acid metabolism	SMPDB	5	17	0.294	2.77E-06	0.0102
Incretin synthesis, secretion, and inactivation	Wikipathways	5	19	0.263	5.10E-06	0.0102
Signal transduction	Reactome	11	184	0.060	3.34E-05	0.0443
Acyl chain remodeling of CL	Reactome	3	6	0.500	5.67E-05	0.0565
Biosynthesis of unsaturated fatty acids	KEGG	5	32	0.156	7.71E-05	0.0615
Free fatty acid receptors	Reactome	4	20	0.200	0.000161	0.1070
Ibuprofen action pathway	SMPDB	6	72	0.083	0.000499	0.1700
Toxicity of tetanus toxin	Reactome	2	3	0.667	0.000626	0.1700
Neurotoxicity of clostridium toxins	Reactome	2	3	0.667	0.000626	0.1700
Anti-inflammatory signaling pathway	BioCarta	2	4	0.500	0.001240	0.1700

Supplementary eTable 3. Clinical characteristics of PD patients with and without depression included in the tandem mass tag-based proteomics analysis.

**Variable (SEM/%)**	**PD (30)**	**PDD (15)**	***p* value**	**Variable (SEM/%)**	**PD (30)**	**PDD (15)**	***p* value**
Age (year)	69.17±1.50	68.20±2.66	0.734	RBG (mmol/L)	6.90±0.37	7.27±0.57	0.595
Gender, Male (%)	19 (63.3%)	6 (40.0%)	0.138	HbA1C (%)	6.17±0.19%	7.10±1.25%	0.513
Smoking history (%)	7 (23.3%)	1 (6.7%)	0.335	TC (mmol/L)	4.32±0.16	3.91±0.18	0.114
Alcohol consumption (%)	1 (3.3%)	0 (0%)	1.000	TG (mmol/L)	1.31±0.14	1.18±0.22	0.589
Hypertension (%)	13 (43.3%)	3 (20.0%)	0.123	HDL-C (mmol/L)	1.32±0.07	1.42±0.08	0.420
Diabetes mellitus (%)	5 (16.7%)	2 (13.3%)	1.000	LDL-C (mmol/L)	2.73±0.13	2.29±0.18	0.056
Hypercholesterolemia (%)	6 (20.0%)	2 (13.3%)	0.890	Apo-A1 (g/L)	1.30±0.05	1.34±0.05	0.610
CHD (%)	7 (23.3%)	3 (20.0%)	1.000	Apo-B (g/L)	0.85±0.04	0.71±0.05	0.049
Disease duration (year)	5.23±0.81	7.07±1.03	0.184	HAMD score	10.40±0.86	20.67±0.51	0.000
BMI (kg/m^2)	22.79±0.58	22.88±0.92	0.929				

PD, Parkinson's disease; SEM, standard error of the mean; PDD, Parkinson's disease related depression; RBG, random blood glucose; HbA1C, hemoglobin A1C; TC, total cholesterol; TG, triglyceride; HDL-C, high-density lipoprotein cholesterol; LDL-C, low-density lipoprotein cholesterol; Apo-A1, apolipoprotein A1; CHD, coronary heart disease; Apo-B, apolipoprotein B; HAMD, Hamilton Depression Scale; BMI, body mass index.

Supplementary eTable 4. The top 20 most enriched GO terms and involved proteins based on proteomics analysis between PD patients with and without depression.

Enriched GO term	Involved protein	*q* value	Pathway impact
Single-organism carbohydrate catabolic process	E7EUT5, P00338, I3L252	0.005	0.150
Carbohydrate catabolic process	E7EUT5, P00338, I3L252	0.006	0.143
Immune effector process	U5YKD2, A0A075B6R9, V9HW68, A0A125U0U7, Q04721, Q53HT9, I3L252	0.014	0.045
Monosaccharide catabolic process	E7EUT5, I3L252	0.016	0.182
Hexose catabolic process	E7EUT5, I3L252	0.016	0.182
Positive regulation of alpha-beta T cell proliferation	U5YKD2	0.018	1
Negative regulation of defense response to virus	I3L252	0.018	1
Regulation of cell cycle G2/M phase transition	Q8NCM2	0.018	1
Regulation of alpha-beta T cell proliferation	U5YKD2	0.018	1
Cytoplasmic pattern recognition receptor signaling pathway in response to virus	I3L252	0.018	1
Negative regulation of MDA-5 signaling pathway	I3L252	0.018	1
Negative regulation of viral-induced cytoplasmic pattern recognition receptor signaling pathway	I3L252	0.018	1
Positive regulation of CD8-positive, alpha-beta T cell proliferation	U5YKD2	0.018	1
Regulation of viral-induced cytoplasmic pattern recognition receptor signaling pathway	I3L252	0.018	1
Glycerone kinase activity	I3L252	0.018	1
Transcription factor activity, ligand-activated RNA polymerase II transcription factor binding	Q04721	0.018	1
T cell receptor binding	U5YKD2	0.018	1
Triokinase activity	I3L252	0.018	1
Anchoring collagen complex	D6RGG3	0.018	1
Golgi medial cisterna	U5YKD2	0.018	1

GO, gene ontology; PD, Parkinson's disease; a shade of yellow color represents GO term from biological process, a shade of green color represents GO term from molecular function, and a shade of red color represent GO terms from cellular component.

Supplementary eTable 5. Enriched KEGG pathways and involved proteins based on proteomics analysis between PD patients with and without depression.

Enriched KEGG pathway	Involved protein	*q* value	Pathway impact
Glucagon signaling pathway	CREB3L3, LDHA	0.005	0.333
Cocaine addiction	CREB3L3	0.018	1.000
RIG-I-like receptor signaling pathway	TKFC	0.018	1.000
Insulin secretion	CREB3L3	0.018	1.000
Viral carcinogenesis	CREB3L3, HLA-A	0.022	0.154
Glycolysis/Gluconeogenesis	LDHA, GAPDH	0.029	0.133
Aldosterone synthesis and secretion	CREB3L3	0.037	0.500
Type I diabetes mellitus	HLA-A	0.037	0.500
Cholinergic synapse	CREB3L3	0.037	0.500
Propanoate metabolism	LDHA	0.037	0.500
Amphetamine addiction	CREB3L3	0.037	0.500
Graft-versus-host disease	HLA-A	0.037	0.500

KEGG, Kyoto Encyclopedia of Genes and Genomes; PD, Parkinson's disease.

Supplementary eTable 6. Significant molecular or cellular function annotations and enriched categories from integrated analysis between PD patients with and without depression.

Molecular or cellular function annotation	*p* value	Included molecules
Consumption of L-arginine	0.00194	1
Catalysis of ethanol	0.00387	1
Formation of histamine	0.00387	1
Concentration of ATP	0.00566	3
Secretion of bilirubin	0.0058	1
Glucuronidation of moxifloxacin	0.00772	1
Release of glucagon	0.00772	1
Synthesis of urea	0.00772	1
Quantity of bilirubin	0.00953	2
Consumption of NADPH	0.00964	1
Synthesis of L-dopa	0.00964	1
Synthesis of arginine	0.00964	1
Binding of melatonin	0.0116	1
Uptake of cimetidine	0.0116	1
Conversion of nitric oxide	0.0135	1
Conversion of L-arginine	0.0154	1
Release of nitrite	0.0154	1
Binding of guanosine 5'-O-(3-thiotriphosphate)	0.0179	2
Synthesis of melatonin	0.0192	1
Synthesis of nucleotide	0.0229	4
Quantity of S-adenosylmethionine	0.0249	1
Uptake of amino acids	0.0305	2
Biosynthesis of cyclic nucleotides	0.034	3
Uptake of L-alanine	0.038	1
Secretion of ATP	0.0455	1
Uptake of methotrexate	0.0154	1
Conversion of citrulline	0.0173	1
Synthesis of citrulline	0.0192	1
Synthesis of D-glucose	0.00151	3
Production of lactic acid	0.00643	2
Transport of D-glucose	0.00691	3
Concentration of sorbitol	0.00964	1
Sulfation of proteoglycan	0.0135	1
Concentration of D-glucose	0.014	4
Oxidation of glucose-6-phosphate	0.0154	1
Quantity of UDP-N-acetylglucosamine	0.0154	1
Processing of N-glycan	0.0211	1
Peroxidation of lipid	2.7E-07	6
Conversion of lipid	6.2E-07	7
Quantity of diacylglycerol	0.00043	3
Concentration of 6-keto-prostaglandin F1 alpha	0.00055	2

Efflux of cholecalciferol	0.00194	1
Localization of lipid	0.00198	2
Synthesis of acylglycerol	0.00232	3
Synthesis of fatty acid	0.00257	5
Synthesis of diacylglycerol	0.00375	2
Binding of galactosylceramide-alpha	0.00387	1
Binding of leukotriene B4	0.00387	1
Esterification of phospholipid	0.00387	1
Loading of triacylglycerol	0.00387	1
Localization of vitamin E	0.00387	1
Synthesis of eicosanoid	0.00395	4
Accumulation of lipid droplets	0.00423	2
Conversion of fatty acid	0.0051	2
Binding of 15(S)-HETE	0.0058	1
Co-localization of ganglioside GM1	0.0058	1
Conversion of 12(S)-hydroxyeicosatetraenoic acid	0.0058	1
Conversion of palmitic acid	0.0058	1
Deacylation of phospholipid	0.0058	1
Uptake of anandamide	0.0058	1
Mass of triacylglycerol	0.00772	1
Quantity of very long chain fatty acid	0.00772	1
Conversion of triacylglycerol	0.00964	1
Localization of triacylglycerol	0.00964	1
Metabolism of palmitic acid	0.0116	1
Oxidation of phospholipid	0.0116	1
Secretion of vitamin E	0.0116	1
Synthesis of triolein	0.0116	1
Production of ketone body	0.0135	1
Concentration of phospholipid	0.0155	3
Concentration of acylglycerol	0.0169	4
Synthesis of lipid	0.021	6
Biosynthesis of stearic acid	0.023	1
Conversion of arachidonic acid	0.023	1
Incorporation of triacylglycerol	0.023	1
Oxidation of lipid	0.0247	3
Conversion of cholesterol	0.0249	1
Deposition of triacylglycerol	0.0249	1
Synthesis of oleic acid	0.0268	1
Binding of lipid	0.0282	2
Quantity of cardiolipin	0.0287	1
Quantity of 2-arachidonoylglycerol	0.0324	1
Synthesis of cholesterol ester	0.0343	1
Synthesis of palmitic acid	0.0362	1
Concentration of lipid	0.0389	6

Concentration of anandamide	0.0399	1
Quantity of leukotriene B4	0.0436	1
Formation of lipid droplets	0.0455	1
Quantity of non-esterified fatty acid	0.0455	1
Storage of triacylglycerol	0.0455	1
Accumulation of cholesterol ester	0.0492	1
Concentration of malonyl-coenzyme A	0.0492	1
Concentration of fatty acid	0.0498	3
Distribution of phosphatidylethanolamine	0.00194	1
Distribution of phosphatidylcholine	0.0058	1
Concentration of phosphatidylcholine	0.00584	2
Quantity of lysophosphatidylcholine	0.0399	1
Quantity of phosphatidylethanolamine	0.0417	1
Production of hydrogen peroxide	0.00166	3
Activation of hydrogen peroxide	0.00772	1
Gluconeogenesis	1E-04	4
Synthesis of monosaccharide	0.00012	4
Gluconeogenesis of hepatocytes	0.00075	2
Synthesis of D-glucose	0.00151	3
Distribution of phosphatidylethanolamine	0.00194	1
Elimination of glyceraldehyde-3-phosphate	0.00194	1
Production of glyceraldehyde-3-phosphate	0.00387	1
Quantity of carbohydrate	0.00469	6
Distribution of phosphatidylcholine	0.0058	1
Concentration of phosphatidylcholine	0.00584	2
Production of lactic acid	0.00643	2
Transport of D-glucose	0.00691	3
Synthesis of carbohydrate	0.0075	5
Concentration of sorbitol	0.00964	1
Sulfation of proteoglycan	0.0135	1
Concentration of D-glucose	0.014	4
Oxidation of glucose-6-phosphate	0.0154	1
Quantity of UDP-N-acetylglucosamine	0.0154	1
Conversion of pyruvic acid	0.0173	1
Processing of N-glycan	0.0211	1
Glycolysis of cells	0.0325	2
Quantity of lysophosphatidylcholine	0.0399	1
Quantity of phosphatidylethanolamine	0.0417	1
Peroxidation of lipid	2.7E-07	6
Conversion of lipid	6.2E-07	7
Quantity of diacylglycerol	0.00043	3
Concentration of 6-keto-prostaglandin F1 alpha	0.00055	2
Distribution of phosphatidylethanolamine	0.00194	1
Efflux of cholecalciferol	0.00194	1

Localization of lipid	0.00198	2
Synthesis of acylglycerol	0.00232	3
Synthesis of fatty acid	0.00257	5
Synthesis of diacylglycerol	0.00375	2
Binding of galactosylceramide-alpha	0.00387	1
Binding of leukotriene B4	0.00387	1
Esterification of phospholipid	0.00387	1
Loading of triacylglycerol	0.00387	1
Localization of vitamin E	0.00387	1
Synthesis of eicosanoid	0.00395	4
Accumulation of lipid droplets	0.00423	2
Conversion of fatty acid	0.0051	2
Binding of 15(S)-HETE	0.0058	1
Co-localization of ganglioside GM1	0.0058	1
Conversion of 12(S)-hydroxyeicosatetraenoic acid	0.0058	1
Conversion of palmitic acid	0.0058	1
Deacylation of phospholipid	0.0058	1
Distribution of phosphatidylcholine	0.0058	1
Uptake of anandamide	0.0058	1
Concentration of phosphatidylcholine	0.00584	2
Mass of triacylglycerol	0.00772	1
Quantity of very long chain fatty acid	0.00772	1
Conversion of triacylglycerol	0.00964	1
Localization of triacylglycerol	0.00964	1
Metabolism of palmitic acid	0.0116	1
Oxidation of phospholipid	0.0116	1
Secretion of vitamin E	0.0116	1
Synthesis of triolein	0.0116	1
Production of ketone body	0.0135	1
Concentration of phospholipid	0.0155	3
Concentration of acylglycerol	0.0169	4
Synthesis of lipid	0.021	6
Biosynthesis of stearic acid	0.023	1
Conversion of arachidonic acid	0.023	1
Incorporation of triacylglycerol	0.023	1
Oxidation of lipid	0.0247	3
Conversion of cholesterol	0.0249	1
Deposition of triacylglycerol	0.0249	1
Synthesis of oleic acid	0.0268	1
Binding of lipid	0.0282	2
Quantity of cardiolipin	0.0287	1
Quantity of 2-arachidonoylglycerol	0.0324	1
Synthesis of cholesterol ester	0.0343	1
Synthesis of palmitic acid	0.0362	1

Concentration of lipid	0.0389	6
Concentration of anandamide	0.0399	1
Quantity of lysophosphatidylcholine	0.0399	1
Quantity of phosphatidylethanolamine	0.0417	1
Quantity of leukotriene B4	0.0436	1
Formation of lipid droplets	0.0455	1
Quantity of non-esterified fatty acid	0.0455	1
Storage of triacylglycerol	0.0455	1
Accumulation of cholesterol ester	0.0492	1
Concentration of malonyl-coenzyme A	0.0492	1
Concentration of fatty acid	0.0498	3
Activation of hydrogen peroxide	0.00772	1
Generation of reactive oxygen species	0.00476	4
Generation of superoxide	0.0158	2
Production of reactive oxygen species	2.4E-07	10
Production of superoxide	3E-05	5
Production of hydrogen peroxide	0.00166	3
Accumulation of reactive oxygen species	0.0183	2
Peroxidation of lipid	2.7E-07	6
Oxidative stress response of liver	0.00061	2
Oxidative stress response of cells	0.011	2
Cytotoxicity of gamma-delta T lymphocytes	0.0116	1
Respiratory burst of peritoneal macrophages	0.0116	1
Cytotoxicity of T lymphocytes	0.014	2
Endoplasmic reticulum stress response of hepatoma cell lines	0.0192	1
Oxidative stress response of hepatocytes	0.0192	1
Injury of cortical neurons	0.0211	1
Oxidative stress response of heart	0.023	1

PD, Parkinson’s disease; a shade of orange color represents annotation categorized into small molecule biochemistry, a shade of green color represents annotation categorized into glucose metabolism, a shade of yellow color represents annotation categorized into lipid metabolism, a shade of red color represents annotation categorized into free radical scavenging, and a shade of bright blue color represents annotation categorized into cellular compromise.
